# Supplementary material for: Environmental DNA gives comparable results to morphology-based indices of macroinvertebrates in a large-scale ecological assessment
Source: PLoS One. 2021 Sep 21;16(9):e0257510. doi: 10.1371/journal.pone.0257510 (PMC8454941; doi:10.1371/journal.pone.0257510)
Supplement: S1 File — (DOCX) [file pone.0257510.s001.docx]

**Supporting Information**

Additional information (S1-S7) supporting the study entitled “Environmental DNA gives comparable results to morphology-based indices of macroinvertebrates in a large-scale ecological assessment.”

Jeanine Brantschen^1,2*^, Rosetta C. Blackman^1,2,3^, Jean-Claude Walser^4^ and Florian Altermatt^1,2,3*^

^1^ Department of Aquatic Ecology, Eawag, Swiss Federal Institute of Aquatic Science and Technology, Duebendorf, Zurich, Switzerland

^2^ Department of Evolutionary Biology and Environmental Studies, Faculty of Science, University of Zurich, Zurich, Zurich, Switzerland

^3^ Research Priority Programme Global Change and Biodiversity (URPP GCB), University of Zurich, Zurich, Zurich, Switzerland

^4^ Department of Environmental Systems Science, Genetic Diversity Center, Federal Institute of Technology, Zurich, Zurich, Switzerland

*Corresponding authors:

E-Mail: [Jeanine.Brantschen@eawag.ch](mailto:Jeanine.Brantschen@eawag.ch) (JB)

E-Mail: [Florian.Altermatt@ieu.uzh.ch](mailto:Florian.Altermatt@ieu.uzh.ch) (FA)

**S1 Table.** Sampling site characteristics for the river sites included in the NAWA monitoring sampled in spring 2019.

| **project** | **site ID** | **drainage area (m^2^)** | **altitude (m.a.S.l.)** | **total volume sampled (ml)** | **nr. of filters** | **kick-net IBCH** | **eDNA IBCH** | **mis**  **classifed** | **canton** | **river** | **location** |
| --- | --- | --- | --- | --- | --- | --- | --- | --- | --- | --- | --- |
| NAWA | CH_002_BS | 977969513 | 250 | 2000 | 4 | 12 | 13.29 | no | BS | Birs | Birskopf |
| NAWA | CH_007_BE | 923592884 | 444 | 2000 | 4 | 15 | 13.19 | no | BE | Emme | Gerlafingen, Steg |
| NAWA | CH_009_SO | 75670119 | 464 | 2000 | 4 | 9 | 13.06 | yes | SO | Limpach | Kyburg |
| NAWA | CH_011_SO | 43827879 | 390 | 2000 | 4 | 16 | 12.74 | yes | SO | Lüssel | Breitenbach |
| NAWA | CH_014_LU | 2257420287 | 430 | 2000 | 4 | 14 | 14.22 | no | LU | Reuss | Luzern, Bahnhof |
| NAWA | CH_015_VS | 909619694 | 659 | 2000 | 4 | 8 | 12.21 | yes | VS | Rhône | Brig |
| NAWA | CH_016_VS | 787171963 | 650 | 2000 | 4 | 14 | 12.63 | no | VS | Vispa | Visp |
| NAWA | CH_017_VS | 3345682125 | 489 | 2000 | 4 | 12 | 13.41 | no | VS | Rhône | Sion |
| NAWA | CH_020_VD | 237051653 | 384 | 2000 | 4 | 13 | 12.68 | no | VD | Venoge | Ecublens, Les Bois |
| NAWA | CH_021_VD | 361757058 | 432 | 2000 | 4 | 11 | 12.28 | no | VD | Thielle | Yverdon, Les Parties |
| NAWA | CH_022_VD | 428906220 | 440 | 2000 | 4 | 12 | 12.92 | no | VD | Broye | Domdidier |
| NAWA | CH_023_SG | 25226837 | 409 | 2000 | 4 | 18 | 13.03 | yes | SG | Steinach | Vor Mündung, Mattenhof |
| NAWA | CH_025_SG | 287823169 | 535 | 2000 | 4 | 15 | 13.57 | no | SG | Sitter | Leebrugg |
| NAWA | CH_026_SG | 690518493 | 475 | 2000 | 4 | 12 | 13.53 | no | SG | Thur | Niederbüren, Golfplatz |
| NAWA | CH_027_SG | 125113337 | 560 | 2000 | 4 | 10 | 13.15 | yes | SG | Necker | Ob Thur, Lütisburg |
| NAWA | CH_028_SG | 90996118 | 495 | 2000 | 4 | 14 | 12.75 | no | SG | Glatt | Niederuzwil, Buechental |
| NAWA | CH_032_AG | 44667675 | 405 | 2000 | 4 | 14 | 13.75 | no | AG | Pfaffnern | Rothrist |
| NAWA | CH_033_AG | 120454426 | 400 | 2000 | 4 | 15 | 14.43 | no | AG | Wyna | Suhr |
| NAWA | CH_034_AG | 118384100 | 380 | 2000 | 4 | 17 | 13.15 | yes | AG | Bünz | Möriken |
| NAWA | CH_035_AG | 66317902 | 335 | 2000 | 4 | 10 | 13.57 | yes | AG | Surb | Döttingen, bei Pegel ALG |
| NAWA | CH_036_AG | 127979200 | 310 | 2000 | 4 | 10 | 13.42 | no | AG | Sissle | Eiken |
| NAWA | CH_039_AG | 246911491 | 380 | 2000 | 4 | 11 | 13.54 | no | AG | Suhre | Suhr, bei Zentrum Bärenmatte |
| NAWA | CH_040_ZH | 2186165750 | 397 | 2000 | 4 | 16 | 13.39 | yes | ZH | Limmat | Hönggersteg |
| NAWA | CH_041_ZH | 403526527 | 358 | 2000 | 4 | 12 | 12.92 | no | ZH | Töss | Freienstein |
| NAWA | CH_042_ZH | 342406774 | 410 | 2000 | 4 | 11 | 12.53 | no | ZH | Sihl | Sihlhölzli |
| NAWA | CH_043_ZH | 417267133 | 339 | 2000 | 4 | 16 | 13.94 | yes | ZH | Glatt | Rheinsfelden |
| NAWA | CH_044_ZH | 165380093 | 436 | 2000 | 4 | 14 | 13.05 | no | ZH | Glatt | Abfluss Greifensee |
| NAWA | CH_045_ZH | 45866644 | 440 | 2000 | 4 | 12 | 12.83 | no | ZH | Aabach | Mönchaltorf |
| NAWA | CH_046_ZH | 62817330 | 441 | 2000 | 4 | 10 | 12.44 | yes | ZH | Aa | Niederuster |
| NAWA | CH_047_ZH | 68086039 | 385 | 2000 | 4 | 13 | 12.41 | no | ZH | Reppisch | Dietikon |
| NAWA | CH_048_ZH | 63669189 | 428 | 2000 | 4 | 9 | 13.29 | yes | ZH | Jona | Nach Rüti |
| NAWA | CH_050_ZH | 1712780793 | 359 | 2000 | 4 | 11 | 13.40 | no | ZH | Thur | Andelfingen, Brücke |
| NAWA | CH_058_BE | 48721920 | 616 | 2000 | 4 | 16 | 13.21 | yes | BE | Chise | Oberdiessbach |
| NAWA | CH_059_BE | 130379901 | 511 | 2000 | 4 | 8 | 13.34 | yes | BE | Gürbe | Vor Mündung, Bodenacher Fähre |
| NAWA | CH_060_BE | 351175659 | 549 | 2000 | 4 | 15 | 13.30 | no | BE | Sense | Thörishaus |
| NAWA | CH_063_BE | 131206035 | 449 | 2000 | 4 | 14 | 14.06 | no | BE | Urtenen | Schalunen |
| NAWA | CH_067_BL | 284669835 | 261 | 2000 | 4 | 14 | 13.60 | no | BL | Ergolz | Augst, Autobahn |
| NAWA | CH_068_JU | 252012900 | 410 | 2000 | 4 | 15 | 12.99 | no | JU | Sorne | Delémont |
| NAWA | CH_069_JU | 72745887 | 465 | 2000 | 4 | 15 | 13.75 | no | JU | Scheulte | Vicques |
| NAWA | CH_070_TG | 213852363 | 391 | 2000 | 4 | 13 | 13.24 | no | TG | Murg | Frauenfeld |
| NAWA | CH_071_TG | 61879270 | 445 | 2000 | 4 | 15 | 13.36 | no | TG | Lauche | Bei Mühle Matzingen |
| NAWA | CH_074_NW | 227953796 | 456 | 2000 | 4 | 11 | 12.91 | no | NW | Engelberger Aa | Oberdorf Ennerberg |
| NAWA | CH_075_ZG | 262697322 | 390 | 2000 | 4 | 15 | 13.44 | no | ZG | Lorze | Frauenthal |
| NAWA | CH_076_ZG | 100335334 | 421 | 2000 | 4 | 16 | 14.20 | no | ZG | Lorze | Letzi |
| NAWA | CH_079_AG | 179148287 | 365 | 2000 | 4 | 10 | 13.87 | yes | AG | Aabach | Niederlenz |
| NAWA | CH_084_JU | 202989500 | 365 | 2000 | 4 | 13 | 12.72 | no | JU | Allaine | Boncourt |
| NAWA | CH_085_NE | 345770383 | 445 | 2000 | 4 | 16 | 14.10 | no | NE | Areuse | Boudry, Brücke |
| NAWA | CH_087_JU | 665897703 | 386 | 2000 | 4 | 14 | 12.83 | no | JU | Birs | Les Riedes-Dessus |
| NAWA | CH_088_JU | 303167009 | 420 | 2000 | 4 | 10 | 13.41 | yes | JU | Doubs | Ocourt, Pesses des Vernes |
| NAWA | CH_089_SO | 235068349 | 404 | 2000 | 4 | 15 | 12.47 | no | SO | Dünnern | Olten, Hammer |
| NAWA | CH_091_GR | 617639084 | 1650 | 2000 | 4 | 12 | 13.02 | no | GR | Inn | S-chanf |
| NAWA | CH_092_BE | 521679032 | 620 | 2000 | 4 | 14 | 13.44 | no | BE | Kander | Hondrich |
| NAWA | CH_093_LU | 481643747 | 432 | 2000 | 4 | 18 | 13.63 | yes | LU | Kleine Emme | Littau-Reussbühl |
| NAWA | CH_094_BE | 214844269 | 430 | 2000 | 4 | 14 | 13.28 | no | BE | La Suze | Biel, Mitte vor Mündung |
| NAWA | CH_098_TI | 754154669 | 200 | 2000 | 4 | 17 | 12.50 | yes | GR | Landquart | Felsenbach |
| NAWA | CH_099_TI | 472533311 | 235 | 2000 | 4 | 9 | 12.24 | no | TI | Maggia | Locarno, Solduno |
| NAWA | CH_100_SZ | 317128899 | 436 | 2000 | 4 | 13 | 12.67 | no | TI | Moesa | Lumino |
| NAWA | CH_101_UR | 819467005 | 445 | 2000 | 4 | 13 | 13.16 | no | SZ | Muota | Wilerbrugg |
| NAWA | CH_106_BE | 1892819339 | 463 | 2000 | 4 | 14 | 13.36 | no | BE | Saane | Marfeldingen |
| NAWA | CH_111_AG | 364323784 | 420 | 2000 | 4 | 14 | 13.48 | no | AG | Wigger | Zofingen |
| NAWA | CH_114_BE | 230888807 | 640 | 2000 | 4 | 10 | 13.20 | no | BE | Emme | Emmenmatt, Mitte |
| NAWA | CH_119_NE | 111787109 | 635 | 2000 | 4 | 13 | 12.89 | no | AI | Sitter | Appenzell |
| NAWA | CH_123_TI | 170932547 | 622 | 2000 | 4 | 14 | 13.37 | no | NE | Seyon | Valangin |
| NAWA | CH_126_VD | 105424203 | 448 | 2000 | 4 | 14 | 13.62 | no | TI | Maggia | Brontallo |
| NAWA | CH_127_VD | 67040227 | 439 | 2000 | 4 | 10 | 12.18 | yes | VD | Talent | Chavornay |
| NAWA | CH_128_VD | 129482855 | 394 | 2000 | 4 | 15 | 12.58 | no | VD | Promenthouse | Le Rancho |
| NAWA | CH_129_VD | 32210434 | 375 | 2000 | 4 | 16 | 13.09 | yes | VD | Boiron Morges | Lac |
| NAWA | CH_130_VD | 93127635 | 395 | 2000 | 4 | 16 | 12.81 | yes | VD | Aubonne | Allaman, Le Coulet |
| NAWA | CH_131_VD | 65077759 | 376 | 2000 | 4 | 9 | 12.49 | yes | VD | Veveyse | Vevey |
| NAWA | CH_132_VD | 144386886 | 386 | 2000 | 4 | 13 | 12.88 | no | VD | Grande Eau | Aigle, Autoroute |
| NAWA | CH_133_BE | 568625065 | 640 | 2000 | 4 | 17 | 12.74 | no | BE | Simme | Latterbach |
| NAWA | CH_134_BE | 258156196 | 475 | 2000 | 4 | 12 | 13.40 | no | BE | La Birse | Choindez-Aval de la Roche St. Jean |
| NAWA | CH_135_AR | 93608234 | 593 | 2000 | 4 | 15 | 14.14 | no | AR | Urnäsch | Kubel, ob Sitter |

S2 Table. List of the taxa at the different taxonomic ranks as used in the IBCH index. The indicator groups are identified to different taxonomic level, to family level at best.

| **Phylum** | **Class** | **Order** | **Family** |
| --- | --- | --- | --- |
| Eukarya | Porifera |  |  |
|  | Cnidaria |  |  |
|  | Bryozoa |  |  |
|  | Platyhelminthes | Dendrocoelidae |  |
|  |  | Dugesiidae |  |
|  |  | Planariidae |  |
|  |  | Planariidae |  |
|  | Nemathelminthes |  |  |
|  | Annelida | Hirudinea |  |
|  |  | Erpobdellidae |  |
|  |  | Glossiphoniidae |  |
|  |  | Hirudidae |  |
|  |  | Piscicolidae |  |
|  |  | Oligochaeta |  |
|  | Mollusca | Gastropoda | Acroloxidae |
|  |  |  | Ancylidae |
|  |  |  | Bithyniidae |
|  |  |  | Ferrissiidae |
|  |  |  | Hydrobiidae |
|  |  |  | Lymnaeidae |
|  |  |  | Neritidae |
|  |  |  | Physidae |
|  |  |  | Planorbidae |
|  |  |  | Valvatidae |
|  |  |  | Viviparidae |
|  |  | Bivalvia | Corbiculidae |
|  |  |  | Dreissenidae |
|  |  |  | Sphaeriidae |
|  |  |  | Unionidae |
|  | Arthropoda | Arachnida | Hydracarina |
|  | Malacostraca | Branchiopoda |  |
|  |  | Amphipoda | Corophiidae |
|  |  |  | Gammaridae |
|  |  |  | Niphargidae |
|  |  | Isopoda | Asellidae |
|  |  |  | Janiridae |
|  |  | Mysida | Mysidae |
|  |  | Decapoda |  |
|  |  | Astacidae |  |
|  |  | Cambaridae |  |
|  | Insecta | Ephemeroptera | Ameletidae |
|  |  |  | Baetidae |
|  |  |  | Caenidae |
|  |  |  | Ephemerellidae |
|  |  |  | Ephemeridae |
|  |  |  | Heptageniidae |
|  |  |  | Leptophlebiidae |
|  |  |  | Oligoneuriidae |
|  |  |  | Polymitarcyidae |
|  |  |  | Potamanthidae |
|  |  |  | Siphlonuridae |
|  |  |  | Odonata |
|  |  |  | Aeshnidae |
|  |  |  | Calopterygidae |
|  |  |  | Coenagrionidae |
|  |  |  | Cordulegasteridae |
|  |  |  | Corduliidae |
|  |  |  | Gomphidae |
|  |  |  | Lestidae |
|  |  |  | Libellulidae |
|  |  |  | Platycnemididae |
|  |  | Plecoptera | Capniidae |
|  |  |  | Chloroperlidae |
|  |  |  | Leuctridae |
|  |  |  | Nemouridae |
|  |  |  | Perlidae |
|  |  |  | Perlodidae |
|  |  |  | Taeniopterygidae |
|  |  |  | Heteroptera |
|  |  |  | Aphelocheiridae |
|  |  |  | Corixidae |
|  |  |  | Gerridae |
|  |  |  | Hebridae |
|  |  |  | Hydrometridae |
|  |  |  | Mesoveliidae |
|  |  |  | Naucoridae |
|  |  |  | Nepidae |
|  |  |  | Notonectidae |
|  |  |  | Pleidae |
|  |  |  | Veliidae |
|  |  | Megaloptera | Sialidae |
|  |  | Neuroptera | Osmylidae |
|  |  |  | Sisyridae |
|  |  | Coleoptera | Curculionidae |
|  |  |  | Chrysomelidae |
|  |  |  | Dryopidae |
|  |  |  | Dytiscidae |
|  |  |  | Elmidae |
|  |  |  | Gyrinidae |
|  |  |  | Haliplidae |
|  |  |  | Helophoridae |
|  |  |  | Hydraenidae |
|  |  |  | Hydrochidae |
|  |  |  | Hydrophilidae |
|  |  |  | Hydroscaphidae |
|  |  |  | Hygrobiidae |
|  |  |  | Noteridae |
|  |  |  | Psephenidae |
|  |  |  | Scirtidae |
|  |  |  | Spercheidae |
|  | Hymenoptera | Trichoptera | Apataniidae |
|  |  |  | Beraeidae |
|  |  |  | Brachycentridae |
|  |  |  | Ecnomidae |
|  |  |  | Glossosomatidae |
|  |  |  | Goeridae |
|  |  |  | Helicopsychidae |
|  |  |  | Hydropsychidae |
|  |  |  | Hydroptilidae |
|  |  |  | Lepidostomatidae |
|  |  |  | Leptoceridae |
|  |  |  | Limnephilidae |
|  |  |  | Molannidae |
|  |  |  | Odontoceridae |
|  |  |  | Philopotamidae |
|  |  |  | Phryganeidae |
|  |  |  | Polycentropodidae |
|  |  |  | Psychomyiidae |
|  |  |  | Ptilocolepidae |
|  |  |  | Rhyacophilidae |
|  |  |  | Sericostomatidae |
|  | Lepidoptera | Diptera | Anthomyiidae |
|  |  |  | Athericidae |
|  |  |  | Blephariceridae |
|  |  |  | Ceratopogonidae |
|  |  |  | Chaoboridae |
|  |  |  | Chironomidae |
|  |  |  | Culicidae |
|  |  |  | Cylindrotomidae |
|  |  |  | Dixidae |
|  |  |  | Dolichopodidae |
|  |  |  | Empididae |
|  |  |  | Ephydridae |
|  |  |  | Limoniidae |
|  |  |  | Psychodidae |
|  |  |  | Ptychopteridae |
|  |  |  | Rhagionidae |
|  |  |  | Scatophagidae |
|  |  |  | Sciomyzidae |
|  |  |  | Simuliidae |
|  |  |  | Stratiomyidae |
|  |  |  | Syrphidae |
|  |  |  | Tabanidae |
|  |  |  | Thaumaleidae |
|  |  |  | Tipulidae |

|  | **name** | **description** | **sequence 5’-3’** | **publication** |  |
| --- | --- | --- | --- | --- | --- |
|  | mICOIintF | forward primer | TGGWACWGGWTGAACWGTWTAYCCYCC | Leray et al., 2013 |  |
|  | jgHCO2198 | reverse primer | TAIACYTCIGGRTGICCRAARAAYCA | Geller et al., 2013 |  |
|  | CO1_dummy | synthetic customised oligo used as positive control | GGAACAGGTTGAACTGTATATCCCCCATCAACCTAGTTACGAAGAGCTATAGATCATATAATCCTTAAGTGGAATGTTAATGTGAGTTCAATATGATACACGCCACAGACTCATGTATGTGGATCGGAAGCCAGCTGTTTCCGACCTCGGAGCCGAGAGTGGTTTCTGAATTACACATGTAAGATAAAATCATTAAAGGTACTAACTCACGAAACCTCAGGATATGCGTGGTTTGCTGAGATTTCTATTTTCTCGTTCTTGATTTAACCACGTAAAATGTGTGAAAACTAAAGGTTCTAGCATTTCTAAGGATCACTACGCCTAACGTCTCACTTTATCTTAAATTTGATTTTTTGGTCACCCTGAAGTTTA |  |  |
|  |  |  |  |  |  |

**S3 Table. Nucleotide sequences of the primers**. The primers were published originally by Leray et al. (2013) for the forward primer and Geller et al. (2013) for the reverse primer. The positive control used in this study: A synthetically generated oligonucleotide sequence inlcuding perfect matching primer sites and in between a random sequence of bases with the average GC content of the target amplicons.

**S4 Table. Ranking of indicator groups based on their proportion by both methods**. Count and read data was square-root transformed to proportions and stacked according their groups. The rank indicates their relative contribution to the gamma diversity of the communities.

|  | **indicator group** | **kick-net rank** | **eDNA rank** |  |
| --- | --- | --- | --- | --- |
|  | Diptera | 1 | 1 |  |
|  | Ephemeroptera | 2 | 2 |  |
|  | Trichoptera | 3 | 4 |  |
|  | Plecoptera | 4 | 3 |  |
|  | Amphipoda | 5 | 6 |  |
|  | Coleoptera | 6 | 9 |  |
|  | Oligochaeta | 7 | NA |  |
|  | Gastropoda | 8 | 8 |  |
|  | Arachnida | 9 | NA |  |
|  | Isopoda | 10 | 7 |  |
|  | Bivalvia | 11 | 9 |  |
|  | Platyhelminthes | 12 | NA |  |
|  | Hirudinea | 13 | 10 |  |
|  | Nemanthelminthes | 14 | NA |  |
|  | Odonata | 15 | 11 |  |
|  | Heteroptera | 16 | NA |  |
|  | Megaloptera | 17 | NA |  |
|  | Branchiopoda | 18 | NA |  |
|  | Cnidaria | 19 | 5 |  |
|  | Decapoda | 20 | NA |  |
|  | Bryozoa | 21 | 13 |  |
|  | Porifera | 22 | 12 |  |

**S5 Fig. Taxonomic richness of indicator groups in Swiss rivers compared between kick-net monitoring and eDNA monitoring.** The richness as number of detected groups does not correlate significantly (adj. R^2^ = 0.026, p = 0.08) between methods. Overall, eDNA detected significantly fewer groups per site (p < 0.001).

**S6 Information.** **Description of the CO1 reference database.**

***Improvement for CO1 reference database***

All OTUs were mapped against a customized taxonomic database for annotation. Therefore, Several sources of CO1 sequences were combined. First, we drew untrimmed references from the MIDORI database (V20180221), where the quality of the assignment depended on the quality of the local alignment (n= 927,386). Second, for the most abundant unassigned OTUs in the data set, we manually added references from NCBI with rigid criteria (100% identity, P-Score > 250). The exact procedure for the assembly of the sequences from NCBI is described by the step-by-step protocol below. Thirdly, we recovered sequences for the target indicator groups of Swiss Ephemeroptera, Plecoptera and Trichoptera from SwissBol if available, or alternatively from NCBI. In total, the reference database contained 929,817 sequences.

Here, we describe the protocol we followed to improve the annotation of the CO1 sequencing data. Abundant unassigned sequences were manually blasted and assigned following those steps:

**1. Identify important/abundant OTUs in data without annotation**

ZOTU3055 k__Eukaryota; p__; c__; o__; f__; g__; s__

**2. Get Fasta Sequence**

For the blast search we have to extract the fasta sequence for the selected ZOTU.

> e_OTU/p571_run200121_COI_ZOTU_c99.fa

>ZOTU3055

CCTTTAAGTGGTATTGAGTTCCATTCTGGTGCTTCTGTTGATTTGGCAATTTTCAGTTTACACCTTTCTGGATTAGGTTC

TTTGTTGGGTGCTATGAATTTTATTACTACTATTTTCAATATGAGAATAGCTAGAATGACTTTGCACAATATGCCATTGT

TTGTTTGGGCTGTTCTTATTACAGCATTTTTGCTTCTTTTATCTATCCCAGTGTTGGCAGGAGGTATTACAATGTTGTTG

ACTGATCGTAACTTCAACACAACTTTCTTCGACCCTGCAGGTGGTGGTGATCCAATTTTATTCCAACACTTGTTTTGGT

**3. Blast ZOTU Sequence**


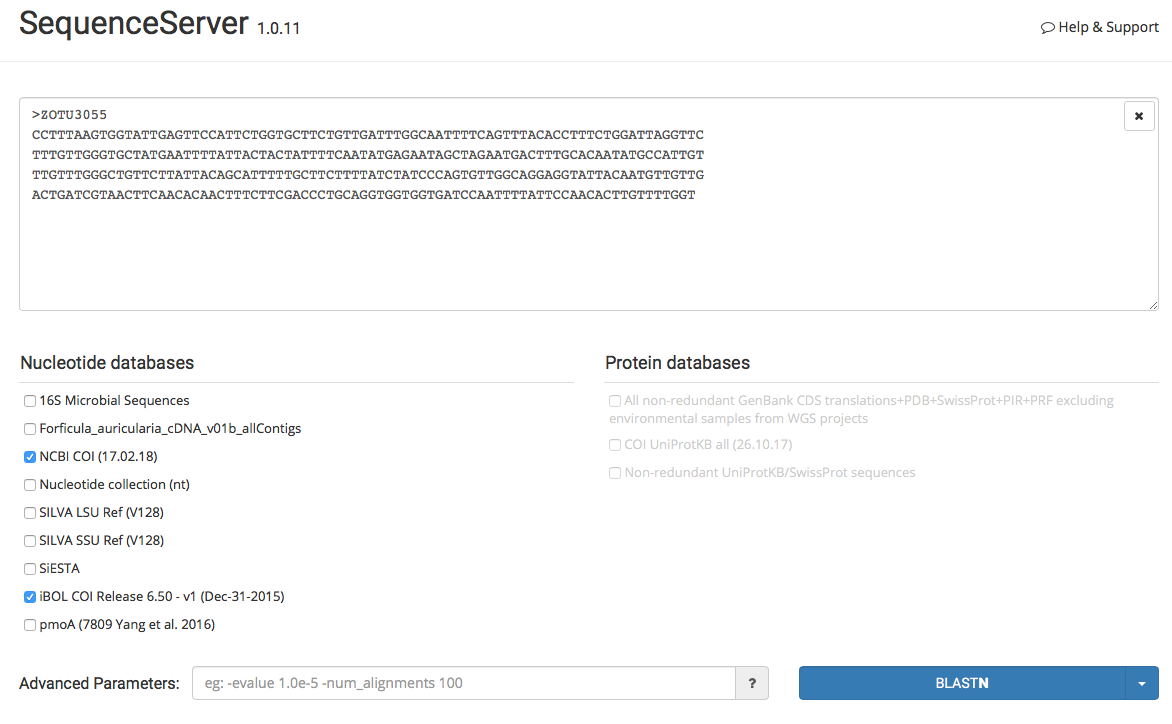


The blast search should not take too long. Once the blast finished, choose good hits with bit-score values > 250. The graphical output shows the length of the coverage with shades of gray indicating quality.

**4. Good Hits**

In this example we have somewhat decent hits.

**
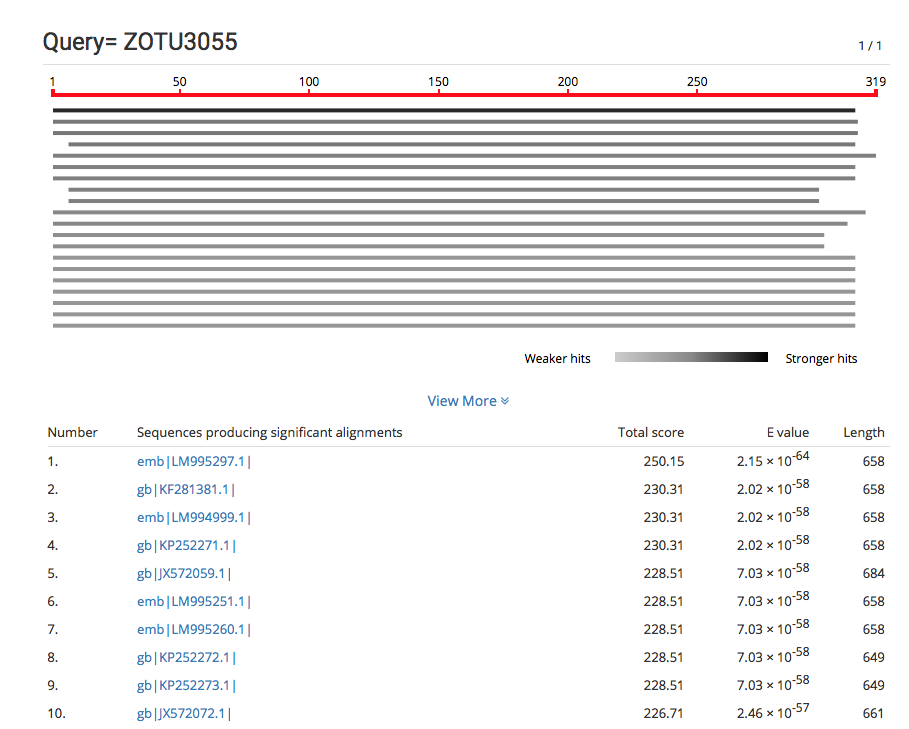
**

Click on the best hits to inspect the alignment. is the best hit (score 250) we have got.


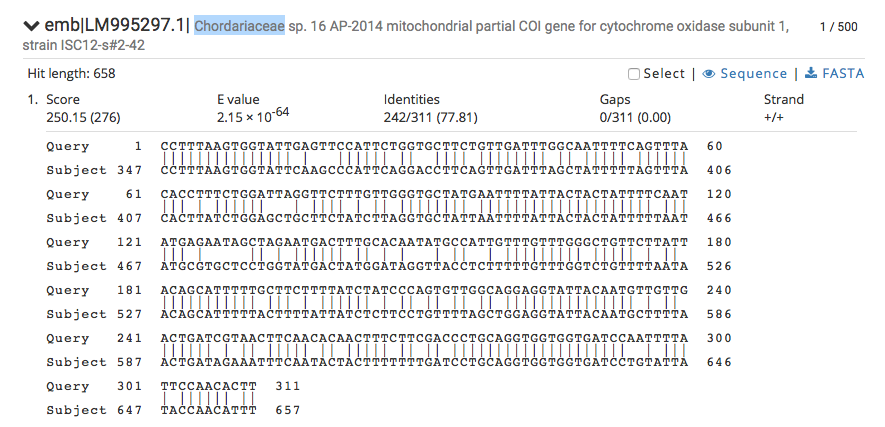


Get the sequence by clicking on [FASTA]. Note: Make sure the alignment is in the +/+ strand direction!

>emb|LM995297.1| Chordariaceae sp. 16 AP-2014 mitochondrial partial COI gene for cytochrome oxidase subunit 1, strain ISC12-s#2-42

TTTATATCTAATCTTTGGTGGTTTCTCTGGAGTATTAGGTACGGCGATGTCTGTTCTTAT

TAGATTGCAATTAGCTAGTCCAGGAAATCAGTTTTTAGGTGGTAATCATCAGTTATATAA

TGTTATTGTTACAGCTCATGCATTTTTAATGATTTTTTTTATGGTTATGCCTGTTCTTAT

TGGAGGATTTGGTAATTGGTTTATACCTTTAATGATTGGTGCTCCTGATATGGCTTTTCC

ACGTATGAATAATATTAGTTTTTGGTTATTACCTCCATCTTTAATACTTCTTTTGGCTTC

TTCTTTAGTAGAGTCTGGTGCAGGTACAGGTTGGACAGTTTATCCACCTTTAAGTGGTAT

TCAAGCCCATTCAGGACCTTCAGTTGATTTAGCTATTTTTAGTTTACACTTATCTGGAGC

TGCTTCTATCTTAGGTGCTATTAATTTTATTACTACTATTTTTAATATGCGTGCTCCTGG

TATGACTATGGATAGGTTACCTCTTTTTGTTTGGTCTGTTTTAATAACAGCATTTTTACT

TTTATTATCTCTTCCTGTTTTAGCTGGAGGTATTACAATGCTTTTAACTGATAGAAATTT

CAATACTACTTTTTTTGATCCTGCAGGTGGTGGTGATCCTGTATTATACCAACATTTA

**5. Trim Sequence**

Trim the sequences according to the alignment.

>emb|LM995297.1| Chordariaceae sp. 16 AP-2014 mitochondrial partial COI gene for cytochrome oxidase subunit 1, strain ISC12-s#2-42

CCTTTAAGTGGTAT

TCAAGCCCATTCAGGACCTTCAGTTGATTTAGCTATTTTTAGTTTACACTTATCTGGAGC

TGCTTCTATCTTAGGTGCTATTAATTTTATTACTACTATTTTTAATATGCGTGCTCCTGG

TATGACTATGGATAGGTTACCTCTTTTTGTTTGGTCTGTTTTAATAACAGCATTTTTACT

TTTATTATCTCTTCCTGTTTTAGCTGGAGGTATTACAATGCTTTTAACTGATAGAAATTT

CAATACTACTTTTTTTGATCCTGCAGGTGGTGGTGATCCTGTATTATACCAACATTT

**6. Replace Header**

We replace the header with a template leaving only the accession number and species label.

>LM995297;tax=d:Eukaryota,p:,c:,o:,f:,g:,s:Chordariaceae_sp.

CCTTTAAGTGGTAT

TCAAGCCCATTCAGGACCTTCAGTTGATTTAGCTATTTTTAGTTTACACTTATCTGGAGC

TGCTTCTATCTTAGGTGCTATTAATTTTATTACTACTATTTTTAATATGCGTGCTCCTGG

TATGACTATGGATAGGTTACCTCTTTTTGTTTGGTCTGTTTTAATAACAGCATTTTTACT

TTTATTATCTCTTCCTGTTTTAGCTGGAGGTATTACAATGCTTTTAACTGATAGAAATTT

CAATACTACTTTTTTTGATCCTGCAGGTGGTGGTGATCCTGTATTATACCAACATTT

7. Complete Header Info

Get the missing label for the taxa level.

As a start we could use NCBI Taxonomy Browser. Maybe not the best choice but at least we all have the same start point.

>LM995297;tax=d:Eukaryota,p:,c:**Phaeophyceae**,o:**Ectocarpales**,f:,g:,s:Chordariaceae_species

CCTTTAAGTGGTAT

TCAAGCCCATTCAGGACCTTCAGTTGATTTAGCTATTTTTAGTTTACACTTATCTGGAGC

TGCTTCTATCTTAGGTGCTATTAATTTTATTACTACTATTTTTAATATGCGTGCTCCTGG

TATGACTATGGATAGGTTACCTCTTTTTGTTTGGTCTGTTTTAATAACAGCATTTTTACT

TTTATTATCTCTTCCTGTTTTAGCTGGAGGTATTACAATGCTTTTAACTGATAGAAATTT

CAATACTACTTTTTTTGATCCTGCAGGTGGTGGTGATCCTGTATTATACCAACATTT

We also do not have the species or the genus name. Since it is the best hit for an important ZOTU and we should not have blanks of NAs in the labels we use specific taxa-label-placeholders.

>LM995297;tax=d:Eukaryota,p:,c:Phaeophyceae,o:Ectocarpales,f:**Chordariaceae**,g:**Chordariaceae_genus**,s:**Chordariaceae_species**

CCTTTAAGTGGTAT

TCAAGCCCATTCAGGACCTTCAGTTGATTTAGCTATTTTTAGTTTACACTTATCTGGAGC

TGCTTCTATCTTAGGTGCTATTAATTTTATTACTACTATTTTTAATATGCGTGCTCCTGG

TATGACTATGGATAGGTTACCTCTTTTTGTTTGGTCTGTTTTAATAACAGCATTTTTACT

TTTATTATCTCTTCCTGTTTTAGCTGGAGGTATTACAATGCTTTTAACTGATAGAAATTT

CAATACTACTTTTTTTGATCCTGCAGGTGGTGGTGATCCTGTATTATACCAACATTT

The label for p is still missing, look on an external source or NCBI taxonomy for the phylum information: Ochrophyta.

>LM995297;tax=d:Eukaryota,p:Ochrophyta,c:Phaeophyceae,o:Ectocarpales,f:Chordariaceae,g:**Chordariaceae_genus**,s:**Chordariaceae_species**

CCTTTAAGTGGTAT

TCAAGCCCATTCAGGACCTTCAGTTGATTTAGCTATTTTTAGTTTACACTTATCTGGAGC

TGCTTCTATCTTAGGTGCTATTAATTTTATTACTACTATTTTTAATATGCGTGCTCCTGG

TATGACTATGGATAGGTTACCTCTTTTTGTTTGGTCTGTTTTAATAACAGCATTTTTACT

TTTATTATCTCTTCCTGTTTTAGCTGGAGGTATTACAATGCTTTTAACTGATAGAAATTT

CAATACTACTTTTTTTGATCCTGCAGGTGGTGGTGATCCTGTATTATACCAACATTT

This is the best hit for an important ZOTU and to avoid NAs in the labels, we use specific taxa-label-placeholders.

**S7 Information**. Amplicon-Sequencing Data Preparation Report for HPC Euler (v100320)

Genetic Diversity Centre (GDC), D-USYS, ETH Zurich / J-C Walser

------------------------------------------------------------------------------------------

Project: p571

Run: run200218

------------------------------------------------------------------------------------------

Project Info

**Locus: COI (Folmer Region)**

**Expected Amp-Size: 313 nt**

**N(sample): 384**

------------------------------------------------------------------------------------------

Run Info (Illumina MiSeq GDC)

------------------------------------------------------------------------------------------

Cluster density: 1109 K/mm2 (Optimal 500–1200 k/mm2)

Reads Total: 26.64 M (goal 25 M)

Reads PF: 24.60 M

PhiX conc: 13.48 % (loaded 10 %)

%>=Q30: Total 87.02 % (should be at least 70 %)

------------------------------------------------------------------------------------------

**Primer Info**

------------------------------------------------------------------------------------------

miCOIintF 5'-GGWACWGGWTGAACWGTWTAYCCYCC-3'

jgHCO2198 5'-TANACYTCNGGRTGNCCRAARAAYCA-3'

================================================================================

**Step A - Data Quality Check**

------------------------------------------------------------------------------------------

Application: usearch v11.0.667_i86linux64

R1: 20,425,546 reads, max len 301, avg 299.1

Lengths min 35, lo_quartile 300, median 300, hi_quartile 301, max 301

Letter freqs T 37.5%, A 25.4%, C 19.4%, G 17.7%, N 0.001%

EE mean 0.7; min 0.0, lo_quartile 0.1, median 0.2, hi_quartile 0.7, max 27.0

Length MaxEE 0.50 MaxEE 1.00 MaxEE 2.00

------ ---------------- ---------------- ----------------

50 20152550( 98.7%) 20402088( 99.9%) 20424824(100.0%)

60 19942881( 97.6%) 20329736( 99.5%) 20420391(100.0%)

70 19723254( 96.6%) 20193758( 98.9%) 20351011( 99.6%)

80 19578412( 95.9%) 20108582( 98.4%) 20317892( 99.5%)

90 19432470( 95.1%) 20023043( 98.0%) 20291018( 99.3%)

100 19264303( 94.3%) 19919024( 97.5%) 20256782( 99.2%)

110 19117095( 93.6%) 19816854( 97.0%) 20219759( 99.0%)

120 18976654( 92.9%) 19719865( 96.5%) 20176992( 98.8%)

130 18877917( 92.4%) 19648518( 96.2%) 20140226( 98.6%)

140 18744453( 91.8%) 19549659( 95.7%) 20089990( 98.4%)

150 18619045( 91.2%) 19456146( 95.3%) 20038864( 98.1%)

160 18416498( 90.2%) 19311121( 94.5%) 19962122( 97.7%)

170 18234418( 89.3%) 19178191( 93.9%) 19887031( 97.4%)

180 18055632( 88.4%) 19044393( 93.2%) 19807865( 97.0%)

190 17910657( 87.7%) 18941325( 92.7%) 19746874( 96.7%)

200 17738724( 86.8%) 18818475( 92.1%) 19671044( 96.3%)

210 17581813( 86.1%) 18699767( 91.6%) 19595072( 95.9%)

220 17410178( 85.2%) 18571610( 90.9%) 19512064( 95.5%)

230 17212029( 84.3%) 18427910( 90.2%) 19419062( 95.1%)

240 17000260( 83.2%) 18275548( 89.5%) 19321848( 94.6%)

250 16705361( 81.8%) 18067620( 88.5%) 19191074( 94.0%)

260 16399166( 80.3%) 17846436( 87.4%) 19047157( 93.3%)

270 16076962( 78.7%) 17613879( 86.2%) 18895187( 92.5%)

280 15627034( 76.5%) 17300855( 84.7%) 18697940( 91.5%)

290 15068990( 73.8%) 16906751( 82.8%) 18445023( 90.3%)

300 12246942( 60.0%) 13965214( 68.4%) 15399238( 75.4%)

R2: 20,425,546 reads, max len 301, avg 299.5

Lengths min 35, lo_quartile 301, median 301, hi_quartile 301, max 301

Letter freqs A 38.1%, T 24.8%, G 19.8%, C 17.3%, N 0.002%

EE mean 2.1; min 0.0, lo_quartile 0.3, median 0.9, hi_quartile 2.5, max 41.7

Length MaxEE 0.50 MaxEE 1.00 MaxEE 2.00

------ ---------------- ---------------- ----------------

50 19308400( 94.5%) 19948379( 97.7%) 20340293( 99.6%)

60 19091059( 93.5%) 19796778( 96.9%) 20253853( 99.2%)

70 18819530( 92.1%) 19594121( 95.9%) 20104579( 98.4%)

80 18618773( 91.2%) 19448628( 95.2%) 20011849( 98.0%)

90 18423413( 90.2%) 19307071( 94.5%) 19925395( 97.6%)

100 18238620( 89.3%) 19164233( 93.8%) 19833633( 97.1%)

110 18063239( 88.4%) 19026985( 93.2%) 19744810( 96.7%)

120 17881027( 87.5%) 18880536( 92.4%) 19644792( 96.2%)

130 17674629( 86.5%) 18719021( 91.6%) 19537553( 95.7%)

140 17477493( 85.6%) 18566713( 90.9%) 19432598( 95.1%)

150 17262021( 84.5%) 18408528( 90.1%) 19330232( 94.6%)

160 17062179( 83.5%) 18257863( 89.4%) 19231194( 94.2%)

170 16837939( 82.4%) 18093620( 88.6%) 19119908( 93.6%)

180 16538359( 81.0%) 17880476( 87.5%) 18983790( 92.9%)

190 16259665( 79.6%) 17684387( 86.6%) 18857631( 92.3%)

200 15878614( 77.7%) 17419538( 85.3%) 18690181( 91.5%)

210 15477389( 75.8%) 17132917( 83.9%) 18507912( 90.6%)

220 14969318( 73.3%) 16769735( 82.1%) 18275803( 89.5%)

230 14441273( 70.7%) 16390545( 80.2%) 18035131( 88.3%)

240 13885159( 68.0%) 15983466( 78.3%) 17771846( 87.0%)

250 13388840( 65.5%) 15614677( 76.4%) 17526511( 85.8%)

260 12543709( 61.4%) 14979545( 73.3%) 17112314( 83.8%)

270 11554067( 56.6%) 14230866( 69.7%) 16619274( 81.4%)

280 10087832( 49.4%) 13069456( 64.0%) 15848386( 77.6%)

290 8823081( 43.2%) 11981572( 58.7%) 15078739( 73.8%)

300 6013133( 29.4%) 8739949( 42.8%) 11644036( 57.0%)

================================================================================

**Step B - Trimming and Merging**

------------------------------------------------------------------------------------------

B1 | Trim Read End

......................................................................

usearch v11.0.667_i86linux64

Trim R1: 60

Trim R2: 120

----------------------------------------------------------------------

B2 | Merge Pairs (Bayesian PE Read Merger)

......................................................................

usearch v11.0.667_i86linux64

Min Overlap: 20

Min %Identity: 70

Min Merged Length: 100

Min Merged Quality: 5

----------------------------------------------------------------------

Median Merged Length: 370

Median Merged Length: 370

------------------------------------------------------------------------------------------

**Step C - Trim Full-Length Primer Sites (in-silico PCR)**

------------------------------------------------------------------------------------------

Application: usearch v11.0.667_i86linux64

Amplicon range: 100-600

Number of mis-matches: 1

Coverage: full-length

Encoding: IUPAC codes

--------------------------------------------------

START_In-Silico_PCR: 14:13:06 15/04/2020

==================================================

END_In-Silico_PCR: 14:43:44 15/04/2020

==========================================================================================

**Step D - Size Selection and Quality Filtering**

------------------------------------------------------------------------------------------

Application: PRINSEQ-lite 0.20.4

Size Range: 300-450

GC Range: 30-70

Min Q Mean: 20

Number of Ns: 0

Low Complexity: dust / 30

------------------------------------------------------------------------------------------

START_QF: 14:44:02 15/04/2020

END_QF: 16:21:37 15/04/2020

------------------------------------------------------------------------------------------

Statistic Report

START_Reporting: 16:21:37 15/04/2020

Sample;Raw;Merged;Primer;Clean;MeanLength

================================================================================

**Step E - Clustering - UPARSE for OTUs & UNOISE for ZOTUs**

----------------------------------------------------------------------

UPARSE : usearch v11.0.667_i86linux64

Min Abundance Size: 2

......................................................................

UNOISE3: usearch v11.0.667_i86linux64

Min Abundance Size: 10

----------------------------------------------------------------------

START_Clustering: 16:33:09 15/04/2020

▶ Deduplicate Amplicons

----------------------------------------------------------------------

Dereplicates amplicons to obtain unique amplicons.

Determin error rates of amplicon reads.

----------------------------------------------------------------------

▶ Cluster OTU (97%)

----------------------------------------------------------------------

Clusters OTU at 97% using the UPARSE-OTU algorithm.

Min Abundance Size: 2

Number of OTUs: 21265

----------------------------------------------------------------------

▶ Unoise3

----------------------------------------------------------------------

Uses the UNOISE algorithm to perform denoising (error-correction) of amplicon reads.

Min Abundance Size: 10

Number of ZOTUs: 15108

----------------------------------------------------------------------

▶ Additional Clustering

----------------------------------------------------------------------

Clusters ZOTUs at different identity levels (i.e. 97%,98% and 99%).

Number of ZOTUs 99%: 7231

Number of ZOTUs 98%: 6304

Number of ZOTUs 97%: 5844

----------------------------------------------------------------------

▶ Count Table

----------------------------------------------------------------------

Generates OTU count tables by mapping reads to OTUs.

----------------------------------------------------------------------

▶ ZOTU Table Report

----------------------------------------------------------------------

Creates a report/summary from an OTU table.

----------------------------------------------------------------------

▶ Octave plots

----------------------------------------------------------------------

Octave plots with low-abundance (Z)OTUs and cross-talk information.

----------------------------------------------------------------------

▶ Alignment and Tree

----------------------------------------------------------------------

▪︎MSA - multiple sequences alignment with MUSCLE

MUSCLE v3.8.1551 by Robert C. Edgar

▪︎CLU - Cluster sequences with usearch.

usearch v11.0.667_i86linux64

Note: The trees will be very approximate in both cases.

----------------------------------------------------------------------

▶ Uncross

----------------------------------------------------------------------

Detects and filters cross-talk (sample mis-assignment) in an OTU table using the UNCROSS algorithm.

----------------------------------------------------------------------

END_Clustering: 09:03:11 16/04/2020

================================================================================

**Step F - Taxonomic Assignment Predictions with SINTAX**

----------------------------------------------------------------------

Application : SINTAX v11.0.667_i86linux64

Reference : Mix COI old + new + EPT + MIDORI (untrimmed)

Reference File: ReferenceG.fa

Tax Filter : 0.85

================================================================================

..................................................

Chimera-Check for OTU: 69

Chimera-Check for ZOTU: 71

Chimera-Check for ZOTU_c99: 29

Chimera-Check for ZOTU_c98: 20

Chimera-Check for ZOTU_c97: 20

End_F6_Chimera-Check: 23:27:57 04/05/2020

--------------------------------------------------

END_Tax-Prediction: 23:27:57 04/05/2020

================================================================================
