# Supplementary material for: Environmental DNA gives comparable results to morphology-based indices of macroinvertebrates in a large-scale ecological assessment
Source: PLoS One. 2021 Sep 21;16(9):e0257510. doi: 10.1371/journal.pone.0257510 (PMC8454941; doi:10.1371/journal.pone.0257510)
Supplement: Attachment — Submitted filename: PONE-D-21-15608_reviewer.pdf [file pone.0257510.s002.pdf]

PLOS ONE

Environmental DNA is comparable to morphology-based indices of macroinvertebrates in a large-scale ecological assessment

--Manuscript Draft--

**Manuscript Number:**	PONE-D-21-15608
**Article Type:**	Research Article
**Full Title:**	Environmental DNA is comparable to morphology-based indices of macroinvertebrates in a large-scale ecological assessment
**Short Title:**	eDNA-based biomonitoring of rivers
**Corresponding Author:**	Jeanine Brantschen<br>Eawag<br>Dübendorf, SWITZERLAND
**Keywords:**	biodiversity, biotic indices, ecological monitoring, machine learning, metabarcoding, water eDNA, water quality
**Abstract:**	Anthropogenic activities are changing the state of ecosystems worldwide, affecting community composition and often resulting in loss of biodiversity. Rivers are among the most impacted ecosystems. Recording their current state with regular biomonitoring is important to assess the future trajectory of biodiversity. However, traditional monitoring methods for ecological assessments are costly and time-intensive. Here, we compare monitoring of macroinvertebrates based on environmental DNA (eDNA) sampling and subsequent metabarcoding with monitoring based on traditional kick-net sampling to assess biodiversity and the biological state of rivers. Both methods were employed to monitor aquatic macroinvertebrate indicator taxa at 92 sites spread across major Swiss river catchments. The metabarcoding data were taxonomically assigned by means of a customized reference database. Only the zero-radius Operational Taxonomic Units assigned to one of the 145 indicator taxa from the kick-net monitoring were used for the comparison of diversity patterns. Overall, we found a strong congruence between both methods for the assessment of overall species richness (gamma diversity), and the same indicator taxa were commonly detected across all sites by both methods. However, when assessing biodiversity at the site level (alpha diversity), the methods were not consistent, and environmental DNA retrieved significantly fewer indicator taxa per site than the kick-net approach. In the next step, we then compared the ecological classification of rivers using these biotic indicator taxa. A random forest approach classified a majority of the predictions (72%) based on eDNA data in the same river status categories as based on the kick-net approach. Thus, the sampling of environmental DNA validly detected indicator communities and, combined with machine learning, provided reliable classifications of the ecological state of rivers. Overall, environmental DNA sampling has the potential to complement traditional surveys of macroinvertebrate communities in routine large-scale assessments in a non-invasive and scalable approach, and its use for calculating ecological indices does not necessarily depend on a one-to-one match at the level of species composition.
**Order of Authors:**	Jeanine Brantschen
	Rosetta Charlotte Blackman
	Jean-Claude Walser
	Florian Altermatt
**Additional Information:**	
**Question**	**Response**
**Financial Disclosure**<br><br>Enter a financial disclosure statement that describes the sources of funding for the work included in this submission. Review	The funders had no role in study design, data collection and analysis, decision to publish, or preparation of the manuscript.

Powered by Editorial Manager® and ProduXion Manager® from Aries Systems Corporation

the submission guidelines for detailed requirements. View published research articles from *PLOS ONE* for specific examples.<br><br>This statement is required for submission and **will appear in the published article** if the submission is accepted. Please make sure it is accurate.<br><br>**Unfunded studies**<br>Enter: *The author(s) received no specific funding for this work.*<br><br>**Funded studies**<br>Enter a statement with the following details:<br>• Initials of the authors who received each award<br>• Grant numbers awarded to each author<br>• The full name of each funder<br>• URL of each funder website<br>• Did the sponsors or funders play any role in the study design, data collection and analysis, decision to publish, or preparation of the manuscript?<br>• **NO** - Include this sentence at the end of your statement: *The funders had no role in study design, data collection and analysis, decision to publish, or preparation of the manuscript.*<br>• **YES** - Specify the role(s) played.<br><br>* typeset	
**Competing Interests**<br><br>Use the instructions below to enter a competing interest statement for this submission. On behalf of all authors, disclose any competing interests that could be perceived to bias this work—acknowledging all financial support and any other relevant financial or non-financial competing interests.<br><br>This statement is required for submission and **will appear in the published article** if the submission is accepted. Please make sure it is accurate and that any funding sources listed in your Funding Information later in the submission form are also	The authors have declared that no competing interests exist.

Powered by Editorial Manager® and ProduXion Manager® from Aries Systems Corporation

declared in your Financial Disclosure statement.<br><br>View published research articles from *PLOS ONE* for specific examples.<br><br>**NO authors have competing interests**<br><br>Enter: *The authors have declared that no competing interests exist.*<br><br>**Authors with competing interests**<br><br>Enter competing interest details beginning with this statement:<br><br>*I have read the journal's policy and the authors of this manuscript have the following competing interests: [insert competing interests here]*<br><br>* typeset	
**Ethics Statement**<br><br>Enter an ethics statement for this submission. This statement is required if the study involved:<br><br>• Human participants<br>• Human specimens or tissue<br>• Vertebrate animals or cephalopods<br>• Vertebrate embryos or tissues<br>• Field research<br><br>Write "N/A" if the submission does not require an ethics statement.<br><br>General guidance is provided below. Consult the submission guidelines for detailed instructions. **Make sure that all information entered here is included in the Methods section of the manuscript.**	N/A

Powered by Editorial Manager® and ProduXion Manager® from Aries Systems Corporation

**Format for specific study types**<br><br>**Human Subject Research (involving human participants and/or tissue)**<br>• Give the name of the institutional review board or ethics committee that approved the study<br>• Include the approval number and/or a statement indicating approval of this research<br>• Indicate the form of consent obtained (written/oral) or the reason that consent was not obtained (e.g. the data were analyzed anonymously)<br><br>**Animal Research (involving vertebrate animals, embryos or tissues)**<br>• Provide the name of the Institutional Animal Care and Use Committee (IACUC) or other relevant ethics board that reviewed the study protocol, and indicate whether they approved this research or granted a formal waiver of ethical approval<br>• Include an approval number if one was obtained<br>• If the study involved *non-human primates*, add *additional details* about animal welfare and steps taken to ameliorate suffering<br>• If anesthesia, euthanasia, or any kind of animal sacrifice is part of the study, include briefly which substances and/or methods were applied<br><br>**Field Research**<br><br>Include the following details if this study involves the collection of plant, animal, or other materials from a natural setting:<br>• Field permit number<br>• Name of the institution or relevant body that granted permission	
**Data Availability**<br><br>Authors are required to make all data underlying the findings described fully available, without restriction, and from the time of publication. PLOS allows rare exceptions to address legal and ethical concerns. See the PLOS Data Policy and FAQ for detailed information.	Yes - all data are fully available without restriction

A Data Availability Statement describing where the data can be found is required at submission. Your answers to this question constitute the Data Availability Statement and **will be published in the article**, if accepted.<br><br>**Important:** Stating 'data available on request from the author' is not sufficient. If your data are only available upon request, select 'No' for the first question and explain your exceptional situation in the text box.<br><br>Do the authors confirm that all data underlying the findings described in their manuscript are fully available without restriction?	
**Describe where the data may be found in full sentences. If you are copying our sample text, replace any instances of XXX with the appropriate details.**<br><br>• If the data are **held or will be held in a public repository**, include URLs, accession numbers or DOIs. If this information will only be available after acceptance, indicate this by ticking the box below. For example: *All XXX files are available from the XXX database (accession number(s) XXX, XXX.*).<br>• If the data are all contained **within the manuscript and/or Supporting Information files**, enter the following: *All relevant data are within the manuscript and its Supporting Information files.*<br>• If neither of these applies but you are able to provide **details of access elsewhere**, with or without limitations, please do so. For example:<br><br>*Data cannot be shared publicly because of [XXX]. Data are available from the XXX Institutional Data Access / Ethics Committee (contact via XXX) for researchers who meet the criteria for access to confidential data.*<br><br>*The data underlying the results presented in the study are available from (include the name of the third party*	All raw Illumina sequencing data files are available from the European Nucleotoide Archive (project number PRJEB44539).

and contact information or URL).<br>• This text is appropriate if the data are owned by a third party and authors do not have permission to share the data.<br><br>* typeset	
Additional data availability information:	Tick here if the URLs/accession numbers/DOIs will be available only after acceptance of the manuscript for publication so that we can ensure their inclusion before publication.

Powered by Editorial Manager® and ProduXion Manager® from Aries Systems Corporation

Manuscript

Click here to access/download;Manuscript;ResearchArticle_Brantschen_etal

Environmental DNA is comparable to morphology-based indices of macroinvertebrates in a large-scale ecological assessment

Short title: eDNA-based biomonitoring of rivers

Author list:

Jeanine Brantschen[1,2,*], Rosetta C. Blackman[1,2,3], Jean-Claude Walser[4] and Florian Altermatt[1,2,3*]

Author list and ORCID ID:

Jeanine Brantschen[1,2,*] - https://orcid.org/0000-0002-2945-3607

Rosetta C. Blackman[1,2,3] - https://orcid.org/0000-0002-6182-8691

Jean-Claude Walser[4] - https://orcid.org/0000-0003-1513-0783

Florian Altermatt[1,2,3*] - https://orcid.org/0000-0002-4831-6958

Affiliations:

[1] Department of Aquatic Ecology, Eawag, Swiss Federal Institute of Aquatic Science and Technology, Duebendorf, Zurich, Switzerland

[2] Department of Evolutionary Biology and Environmental Studies, Faculty of Science, University of Zurich, Zurich, Zurich, Switzerland

[3] Research Priority Programme Global Change and Biodiversity (URPP GCB), University of Zurich, Zurich, Zurich, Switzerland

[4] Department of Environmental Systems Science, Genetic Diversity Center, Federal Institute of Technology, Zurich, Zurich, Switzerland

*Corresponding authors:
E-Mail: Jeanine.Brantschen@eawag.ch (JB)
E-Mail: Florian.Altermatt@ieu.uzh.ch (FA)

Keywords: biodiversity, biotic indices, ecological monitoring, machine learning, metabarcoding, water eDNA, water quality

Number of Words: 7926
Number of Figures: 6

Abstract

Anthropogenic activities are changing the state of ecosystems worldwide, affecting community composition and often resulting in loss of biodiversity. Rivers are among the most impacted ecosystems. Recording their current state with regular biomonitoring is important to assess the future trajectory of biodiversity. However, traditional monitoring methods for ecological assessments are costly and time-intensive. Here, we compare monitoring of macroinvertebrates based on environmental DNA (eDNA) sampling and subsequent metabarcoding with monitoring based on traditional kick-net sampling to assess biodiversity and the biological state of rivers. Both methods were employed to monitor aquatic macroinvertebrate indicator taxa at 92 sites spread across major Swiss river catchments. The metabarcoding data were taxonomically assigned by means of a customized reference database. Only the zero-radius Operational Taxonomic Units assigned to one of the 145 indicator taxa from the kick-net monitoring were used for the comparison of diversity patterns. Overall, we found a strong congruence between both methods for the assessment of overall species richness (gamma diversity), and the same indicator taxa were commonly detected across all sites by both methods. However, when assessing biodiversity at the site level (alpha diversity), the methods were not consistent, and environmental DNA retrieved significantly fewer indicator taxa per site than the kick-net approach. In the next step, we then compared the ecological classification of rivers using these biotic indicator taxa. A random forest approach classified a majority of the predictions (72%) based on eDNA data in the same river status categories as based on the kick-net approach. Thus, the sampling of environmental DNA validly detected indicator communities and, combined with machine learning, provided reliable classifications of the ecological state of rivers. Overall, environmental DNA sampling has the potential to complement traditional surveys of

macroinvertebrate communities in routine large-scale assessments in a non-invasive and scalable approach, and its use for calculating ecological indices does not necessarily depend on a one-to-one match at the level of species composition.

Introduction

Human activities change natural habitats and thereby inherently affect biodiversity [1]. Freshwater ecosystems are among the most affected and are facing steep biodiversity declines due to anthropogenic pressures [2]. To quantify changes in diversity and community structure of natural communities, monitoring of species is essential [3]. Over decades, ecologists have built an understanding of the responsiveness of certain taxonomic groups to pressures based on assemblages of communities and changes therein [4]. The ecological knowledge of species allows for the interpretation biodiversity pattern to assess environmental pressures and impacts on ecosystems. Routine biomonitoring often classifies ecosystem states through biotic indices that succinctly summarize the information of species assemblages, allowing for comparison to reference states or systems [5,6].

The classification of ecological integrity based on biotic indices is generally focused on certain taxonomic groups. In freshwater ecosystems, the most commonly used are fish, macroinvertebrates, macrophytes, and diatoms [7,8]. For these groups, monitoring generally involves the capture, preservation, and morphological identification of specimens in the field or laboratory. This type of monitoring can be costly in terms of time and money, requiring expert taxonomic skills and often missing rare, small, or elusive species [9].

In the last decade, molecular approaches have proven effective for the assessment of distributions of individual species (species-specific) and community assemblages (metabarcoding approaches). Thereby, DNA extracted from an environmental sample (so-called environmental DNA, eDNA) provides information about the possible occurrence and

distribution of species [10–13]. Monitoring based on eDNA sampling has been explored for different indicator taxa, including fish (e.g., [14,15]); macroinvertebrates (e.g., [16–18]); macrophytes (e.g., [19,20]) and diatoms (e.g., [21,22]). In several instances, eDNA sampling was shown to complement traditional approaches for the assessment of biological indicators and the ecological state of ecosystems [23–25]. Most comparisons of monitoring indicator taxa have strongly focused on reproducing diversity patterns observed by traditional approaches and have paid less attention to possible challenges of eDNA-based metabarcoding.

As eDNA and kick-net sample fundamentally different units (DNA vs. specimen), the processing of samples and the interpretation of the detection cannot be compared one-to-one. The community recovered from an eDNA sample strongly depends on hydrological conditions of the waterbody (e.g., transport of DNA) and choices in the downstream processing (e.g., the barcoding regions, primer choice). Contrastingly, traditional monitoring employs morphologically identifiable indicator organisms, thereby often using a subset of species as a para- or even polyphyletic group (e.g., [26]). For example, the widely used organisms belonging to the "macroinvertebrates" are defined by their size and function, and not by their phylogenetic unity. Targeting the genetic material of these phylogenetically dispersed organisms therefore aims for the coverage of a large portion of metazoans. It is well known that eDNA-based metabarcoding using generic primers (e.g., targeting Cytochrome Oxidase I) often result in limited taxonomic resolution and extensive amplification of non-target groups [27] such as rotifers or other small eukaryotes [28]. Technical biases of the metabarcoding data (e.g., PCR bias, sequencing errors) restrict the comparability to traditional count data [29,30] and limit the implementation in frameworks of existing biotic indices. Novel approaches to fully exploit and interpret molecular data are

thus in demand [31,32]. A promising approach in the era of big data, machine learning has emerged for the analysis of high-dimensional and complex data [33] and recent studies have demonstrated the use of these approaches, such as random forest model [34], also in an ecological context (e.g., [35–38]). Such approaches may overcome the limitations of trying to match traditional and novel monitoring methods in a one-to-one manner and help to explore the opportunities, but also differences, between both approaches.

Here, we used eDNA metabarcoding for the detection of macroinvertebrate indicator taxa in a large-scale ecological assessment and addressed to which degree diversity pattern and the respective ecological index shows convergence. Kick-net and eDNA samples collected within the biomonitoring program for Swiss surface waters were used i) to compare community richness and composition estimates based on morphological identification versus eDNA metabarcoding data using the same pre-defined taxonomic groups, and ii) to evaluate how a supervised machine learning approach can be used for the prediction of the ecological state of rivers when also incorporating data on taxonomic groups not considered by the traditional approaches. We specifically focus on highly replicated and representative samplings based on a nationwide monitoring scheme run by the Swiss Federal Agency for the Environment, in order to cover all major river systems in Switzerland and to directly provide stakeholder-relevant conclusions.

Methods

Sample collection

The Swiss Federal Office for the Environment (FOEN) carries out routine monitoring of freshwater quality in Switzerland ("Nationale Beobachtung Obergewässerqualität", hereafter: NAWA). The goal of NAWA is to gather long-term reference data of the ecological state of riverine systems. Approximately 100 sites distributed over the major catchments in

Switzerland are monitored regularly. The sampling scheme involves physicochemical parameters and the assessment of biological indicators (fish, macroinvertebrates, macrophytes, and diatoms), whereby macroinvertebrates are sampled using a standardized kick-net approach with subsequent morphological identification of species (see also [39]). In 2019, along with standard kick-net sampling, water samples for eDNA analyses were also taken at 92 of these sampling sites and were analyzed amplifying a barcode region for macroinvertebrates (Fig 1).

Fig 1: Description of the study design. A) The taxonomic composition of benthic macroinvertebrates at each sampling site was assessed with two methods: kick-net and eDNA sampling. Subsequently, the focal index on the biological state (IBCH Index) was calculated from kick-net and eDNA data. B) Map of Switzerland showing the spatial setup of the biomonitoring sampling sites. Sampling sites are given as black points overlaid on the main network of rivers and lakes. Major catchments of Switzerland are highlighted by different blue shading.

In brief: eDNA samples were collected in four replicates before the kick-net sampling. For each sampling site, two filter replicates were taken per riverbank (right and left bank, respectively, total n per site = 4). Water was filtered on site, using a disposable sterile 60 ml syringe and Sterivex filters with a 0.22 µm pore size (Merck Millipore, Merck KgaA, Darmstadt, Germany). A total volume of 2 L was filtered per sampling site. Sterivex filters were then sealed with Luer caps (Merck Millipore, Merck KgaA, Darmstadt, Germany), put in a labeled plastic bag and placed in a cool box for short-term storage. After transport to the lab, filters were stored in the fridge at –20 °C until further processing. After placing the eDNA

samples in the cool box, kick-net samples were collected upstream of the eDNA sampling sites following standard procedures for the sampling of river systems [40]. The traditional sampling of macroinvertebrate groups by contractors followed commonly used kick-net methods [41]. In brief: At each site, the benthic fauna of eight microhabitats were sampled with a kick-net, each microhabitat was sampled for 30 seconds by disturbing the substrate by foot. Coarse organic material, sediment and gravel, and non-target organisms such as fish or amphibians were removed from the sample on site. The remaining sample was preserved with 95 % ETOH on site. In the laboratory, macroinvertebrate specimens were sorted and classified into 145 pre-defined taxonomic indicator groups (S1 Table). These groups were mostly at the family level, except for Porifera, Bryozoa, and Cnidaria, which were only grouped at the phylum level.

eDNA sample processing

The extraction of the eDNA samples took place in a specialized laboratory following clean lab procedures [42]. The DNA was extracted by using the Qiagen PowerWater Sterivex Extraction Kit (Qiagen, Germany) following the manufacturer's protocol. Extractions took place in batches of twelve samples. Field filter controls served as negative extraction controls and were extracted randomly among the samples. The extracted DNA was eluted in 100 µl elution buffer and stored at –20 °C until further processing.

Library preparation

Samples were amplified using a 313 bp fragment of the COI marker. The primer pair used was mICOIintF and jgHCO2198 [43,44] with a modification to include the Nextera® transposase sequences. All samples, negative and positive controls (the latter being a synthetic oligo see S2 Table) were randomized over four 96-well PCR plates. The first PCR was carried out in a total volume of 25 µL containing polymerase AmpliTaq Gold 360° (1.25

U/µL), 0.5 µM each of each primer, 1x Buffer I (Thermo Fisher Scientific, MD, USA), BSA (0.1 mg/µL), dNTP (0.2 mM), MgCl2 (1 mM), SigmaFree water and 2 µL of DNA template was added per reaction. The PCRs were performed on a thermal cycler (Biometra T1 Thermocycler, Analytik Jena GMBH, Ge) using the following touchdown protocol: initial denaturation at 95 °C for 10 min, the first 25 cycles started with the denaturation at 95 °C for 15 s, annealing at 62 °C for 30 s, followed by extension at 72 °C for 30 s. After this the cycler performed 16 cycles where the annealing temperature was reduced by one degree each cycle, performing the last cycle at a temperature of 45 degrees. Final extension was performed at 72 °C for 5 min before the plates were cooled down to 10 °C. All samples were tested for amplification success with the AM320 method on the QiAxcel Screening Cartridge (Qiagen, Germany). First step PCR products were cleaned with the ZR DNA Sequencing clean-up Kit (Zymo Research, USA) following the manufacturer's protocol with the minor modification by which the elution step was prolonged to 2 min. at 4000 g.

The clean amplicons were indexed using the Illumina Nextera XT Index Kit A and D following the manufacturer's protocol (Illumina, Inc., San Diego, CA, USA). A reaction contained 25 µL 2x KAPA HIFI HotStart ReadyMix (Kapa Biosystems, Inc., USA), 5 µL of each of the Nextera XT Index adaptors, and 15 µL of the DNA templates. The second reaction had the following PCR protocol: initial activation at 95 °C for 10 min, thermal cycling following a denaturation at 95 °C for 30 s; annealing at 55 °C for 30 s; extension 72 °C for 30 s. After 8 cycles of final extension at 72 °C for 5 minutes, they were cooled to 10 °C and stored in the fridge at 4 °C for downstream application. PCR products were then cleaned using the Thermo MG Magjet bead clean-up kit and a customized program for the KingFisher Flex Purification System (Thermo Fisher Scientific Inc., MA, USA) to remove excessive Nextera XT adaptors. The cleaned product was then eluted 50 µl in a fresh plate and stored at 4 °C.

DNA quantification and normalization

Clean PCR products were measured using the Qubit BR DNA Assay Kit (Life Technologies, Carlsbad, CA, USA). DNA of samples and the standard dilution series were quantified in replicates on a Spark Multimode Microplate Reader (Tecan, US Inc., USA). Samples were united in a normalization step into equimolar pools, according to their respective concentration using the BRAND Liquid Handling Station (BRAND GMBH + CO KG, Wertheim, GE). Negative extraction and PCR controls were added according to their concentration. The final library was cleaned using SPRI beads (0.8 x) twice. Library concentration was quantified by the Qubit the HS Assay Kit and amplicon size was verified on the Agilent 4200 TapeStation (Agilent Technologies, Inc., USA). The Nextera XT library prep Kit (Illumina, Inc. San Diego, CA, USA) was used before loading the library onto the flow cell with a 16 pM target concentration and 10% PhiX. Paired-end sequencing using v3 chemistry was performed on an Illumina MiSeq (Illumina, Inc. San Diego, CA, USA) at the Genetic Diversity Center (ETH, Zurich).

Bioinformatic amplicon sequence analysis and quality filtering

The bioinformatics workflow for post-sequencing data processing used the following approach: the data quality of the demultiplexed reads was checked using FastQC [45]. Raw reads were first end-trimmed; merged and full-length primer sites removed using usearch (v11.0.667_i86linux64) [43]. The merged and primer trimmed reads were quality filtered using prinseq-lite (v0.20.4). The UNOISE3 (usearch v10.0.240) [44] method with an additional clustering at 99% identity was applied to obtain error corrected and chimera-filtered sequence variants (zero-radius OTUs) [45]. The invertebrate mt code was used to check for stop codons in sequences; retained were zOTUS (OTUs hereafter) with open reading frames. These OTUs were mapped against a customized COI reference for taxonomic

assignments (S5). A more detailed workflow report file including parameter settings and documenting data loss are specified in the Supporting Information (S6).

Statistical analysis

All analyses were performed in R (v3.6.0) [46]. The data were imported using the package (v1.28.0) [47]. In the first step, raw data were filtered based on the detection of OTUs in negative and positive controls. For this, a read threshold of OTU detected in controls was calculated and subsequently subtracted from every sample. Samples with low amplification were excluded from further analysis. To reduce spurious and stochastically detected OTUs, the filter replicates per site were used to establish stringency filters. With the least stringent filter, an OTU had to be detected in 1 out of the 4 filters to be retained. Increasing stringency kept only OTUs detected in at least 2, 3, or 4 out of 4 filter replicates in the data. For further analysis, the data with an OTU detection of 2 out of 4 filters were used.

To compare the detection of indicators from the kick-net and the eDNA data, a taxonomic filter removed non-target OTUs as defined by the monitoring framework (S1 Table).

After those filter steps, spatial pattern of indicator group richness was plotted using Swissriverplot (v1.28.0) [48]. In a second step, the taxonomic composition was compared using ggalluvial (v0.12.0) [49] and ggplot2 (v3.3.2) [50]. For eDNA metabarcoding data, the proportion of a taxon was calculated as "reads of all OTUs within one indicator group / total number reads". For kick-net-samples, proportions are "counts within one indicator group / total number of counts".

Under the framework of the Swiss-wide NAWA biomonitoring, the ecological state of rivers is evaluated based on the calculation of a biotic index from kick-net data [39]. The index accounts for taxa diversity and taxa indicator values and is represented as a numerical score from 0 to 20. The higher the score, the less anthropogenic influence is recognizable at a site.

The numerical scores are then categorized into five categories "bad" to "very good". For the calculation of the index, specimens captured in kick-net samples had been sorted, identified to phylum or family level (S1 Table), and counted. The calculation is based on the diversity and indicator value of the taxa [41]. The eDNA data were also analyzed to predict the biotic index, using a supervised random forest model. In order to run the random forest model, we included only indicator macroinvertebrate groups, therefore we subset the eDNA data to the OTUs assigned to the following phyla: Arthropoda, Cnidaria, Porifera, Bryozoa, Mollusca. The presence-absence data of OTUs were used to train a random forest model based on a taxonomy-free approach [36]. A grid search was performed in caret (v6.0.86) [51] to establish the optimal value for the parameters mtry (n/3), node size (3), and ntrees (500). A random forest model with those optimal parameters was fitted for every sample using ranger (v0.12.1) [52]. Based on a random subset of the samples (mtry = n/3), the random forest classifier was trained using OTUs of indicator taxa as predictive features and the index score calculated from kick-net as the response variable. The classifier subsequently predicted the biotic index of a sample based on the OTU composition. This biotic index was predicted for every sample iteratively in ranger. The relationship between the observed and the predicted score was evaluated based on the adjusted R^2 of a linear model and the goodness of fit based on Cohen's Kappa κ [53]. The level of agreement is described for all values as: $\kappa < 0.05$: no agreement, $0.05 < \kappa < 0.20$: very poor, $0.20 < \kappa < 0.40$: poor, $0.40 < \kappa < 0.55$: fair, $0.55 < \kappa < 0.70$: good, $0.70 < \kappa < 0.85$: very good, $0.85 < \kappa < 0.99$: excellent, and $\kappa = 1$: perfect.

Results

Summary of raw sequencing data

Next generation sequencing of eDNA generated 26.64 million reads of which 24.60 million passed the quality filter. After bioinformatic processing, 15.8 million reads were left for downstream analysis. The average sequencing depth per sampling site (4 filter replicates pooled) was 166,827 reads (range: 30,543 –660,048), covering in total 7,231 OTUs. After removal of weak samples and cleaning of the raw data based on positive and negative controls, only OTUs detected in at least 2 out of 4 filter replicates per sampling site were retained in the data. This step decreased the mean read depth per sampling site to 147,913 reads (range: 40,891–602,621; Fig 2A) and the mean number of OTUs per sampling site to 835 (range: 118–1698; Fig 2B). In total 4,599 OTUs were retained after this "2 out of 4" filter step, and of those, 205 OTUs were assigned to the 145 taxonomic levels of indicator groups used for the calculation of the biotic index. The read abundance distribution indicates that the indicator OTUs generally have a relatively high read coverage but were also interspersed by non-target OTUs (Fig 2C). In the kick-net sampling, a total of 145 possible indicator taxa were assessed (S1 Table), of which 98 were detected in this monitoring campaign.

Fig 2: Filtering of raw sequencing data. Distribution of A) mean read number per sampling site (x 103) and B) mean OTU number per sampling site using thresholds based on detection rate in the four field filter replicates. The detection rate is given with increasing stringency: detection of an OTU in at least 1, 2, 3, or 4 out of 4 filter replicates per site, respectively. C) Read abundance distribution of OTUs (n=4599) detected in at least 2 out of 4 replicates per site. Read abundances of OTUs that were taxonomically assigned to indicator taxa are highlighted in green.

Local diversity pattern (alpha diversity)

To compare the alpha diversity pattern of macroinvertebrates from kick-net with eDNA sampling, indicator group richness was mapped for all 92 sampling sites in Switzerland (Fig 3). The observed mean richness at a site derived from kick-net was 23 indicator taxa (range: 11–41). Constraining the sequencing data to OTUs assigned to all possible indicator taxa, the eDNA approach led to the detection of 205 OTUs in 21 families (OTUs assigned to family, genus or species levels). A large portion of OTUs was not considered for the downstream analysis, either belonging to non-indicator taxa or lacking taxonomic assignments. The observed mean richness of indicators by eDNA sampling was 9 indicator taxa (range: 2–18, Fig 3). A linear model showed a weak correlation of the local richness pattern (adj. R^2 = 0.026, $p = 0.08$) detected by the two methods (S4 Figure). However, eDNA detected significantly fewer indicator taxa at a site level ($p < 0.001$).

Fig 3: Spatial richness pattern. The taxonomic richness of indicator groups in Swiss rivers at each sampling site based on A) kick-net monitoring and B) eDNA monitoring. For the latter, only macroinvertebrates also considered in the traditional biological assessment are included. The color gradient is adjusted to the respective range of indicator richness values.

Overall diversity pattern (gamma diversity)

The overall composition of indicator taxa (gamma diversity) detected by the two methods corresponded adequately for common groups (i.e., Diptera, Ephemeroptera, Plecoptera, Amphipoda). Abundances of indicators were rendered comparable by using read and count data on a log-transformed proportional scale (Fig 4). Abundant indicator taxa in the kick-net sampling were also identified as common groups derived from eDNA. The most frequently

detected indicator taxa were Diptera, Ephemeroptera, Plecoptera, Trichoptera, and Amphipoda. Of those, Diptera and Ephemeroptera ranked equally in kick-net and eDNA data. Proportionally, Trichoptera was more abundant than Plecoptera in kick-net samples, but this ranking was reversed in eDNA data. For less frequently observed groups in the community, their relative rank varied between the two methods (e.g., Cnidaria, Isopoda, Coleoptera, Oligochaeta, Mollusca, or Gastropoda) (S3 Table).

Fig 4: Overall diversity pattern. Proportions of indicator groups detected by kick-net versus eDNA monitoring. The stacked bars indicate proportions of indicator groups inferred by the two methods (proportion of counts for kick-net and of reads for eDNA). For the most common indicator groups, names are given. The flows between the two stacked bars connect families within indicator groups between the methods. A change in flow width indicates a change in proportion depending on the method used.

Inference of the biotic index from eDNA data

The biotic index is calculated based on two components: one is the taxa richness, the other one is the occurrence of indicators groups. Kick-net and eDNA sampling methods picked up diverging richness patterns of indicator taxa, but largely showed a similar composition of indicator communities. The realized biotic index score was on average 13.3 (range: 8–17). The random forest model predictions were highly correlated with the biotic index scores observed from kick-net sampling ($R^2 = 0.61$, $p < 0.001$) (Fig 5).

Fig 5: Biotic index based on bioindicators. Comparison of the index on the biological state (IBCH index) based on kick-net-derived scores (IBCH index observed) versus the predicted

index derived from eDNA data. The predictions are the output from a random forest model deriving IBCH index scores using OTU presence-absence as input. A linear regression model gives the relationship between observed and predicted values (adjusted $R^2 = 0.61$, p-value < 0.001). The colored boxes summarise the numerical index scores ranging from 5 to 20 into categories ranging from "unsatisfactory" to "very good".

Similar to the kick-net assessment, most predictions of the index were centered on the categories "intermediate" to "good", and only a few were predicted to fall into the categories "bad" or "very good" (Fig 6A). The majority of the predictions (72 %) classified the ecological state of sampling sites correspondingly to the traditional kick-net-based estimates, and maximally diverged by one category (Fig 6B).

Fig 6: Distribution of predicted classifications of the biotic state. Comparison of the biological state of sampling sites when comparing classifications based on kick-net or random forest predictions. A) The density distributions for the observed (kick-net-based, grey) and the predicted (eDNA-based, green) IBCH index scores. The x-axis indicates the range of the biological index from 5 to 20. B) Barplot showing the percentage of sites that fell in the same ($x = 0$) or different ($x \neq 0$) category by the random forest predictions based on eDNA data compared to the kick-net-based classifications. The majority of sampling sites were classified in the same category (72 %). All other sites (28 %) were maximally deviating by one category.

Discussion

Molecular surveys for traditionally established bioindicators

Despite the demonstration of eDNA for the survey of species and communities in aquatic ecosystems [10–13], routine implementation of molecular approaches in biomonitoring such as water quality assessments remains scarce. Here, we demonstrated the utility of water eDNA sampling for the assessment of macroinvertebrates on a national scale with a comparison to the traditional kick-net approach. Overall, the most common indicator taxa detected by eDNA were equally well covered by kick-net sampling at the national level (gamma diversity). However, local richness patterns (alpha diversity) were less consistent between the two methods, as eDNA samples on average detected fewer indicator taxa at the site level. Nevertheless, the composition of indicator OTUs at a site effectively informed about the ecological state, when using a machine learning algorithm. This study shows that eDNA is a valuable resource for the detection of macroinvertebrate indicator taxa and subsequent calculation of biotic indices, and it can be implemented for the ecological assessment of rivers.

Local and overall diversity patterns

Diversity patterns of macroinvertebrate communities in this study were restricted to the indicator groups as defined by the 145 taxonomic levels in the biotic IBCH index only. Significantly fewer indicator taxa were detected by eDNA sampling on a site level, although the ranking of the indicator taxa based on their overall relative abundance (proportions) in the community was similar for the most common groups. Diversity assessments of macroinvertebrate communities through COI metabarcoding have often reported similar or higher numbers of taxa compared to traditional methods (e.g., [17,18,54]), however, only a proportion of all reads are assigned to macroinvertebrates and even less to indicator taxa

[55,56]. Arthropods are essential to the biotic assessment based on macroinvertebrates, and were the most dominant group among the OTUs assigned to bioindicators. These were consistently detected by eDNA and kick-net, with Diptera as the most common order, followed by orders with high indicator values, namely Ephemeroptera, Plecoptera, Trichoptera. However, some target groups were starkly underrepresented by eDNA, namely Hemiptera, Arachnida, and Coleoptera. The ecology of the target species delivers a possible explanation, e.g., their hydrophobic exoskeletons and the lower DNA shedding rates of these organisms [57], impairing these species' detection [58].

In river systems, DNA fragments are transported with the water flow [59,60] and thus the detection of local community assemblages is biased, as the DNA signal of species physically present upstream will be integrated into the local sample [61]. The detection probability of target taxa is thus considerably influenced by the sampling strategy [62,63], and should generally consider the dendritic network structure of rivers [64]. Thus, an eDNA sample integrates biodiversity pattern across a larger spatial scale [12] compared to traditional sampling, which to some level may inherently result in mismatches of local richness or identity of organisms measured with this method compared to the kick-net approach.

Another factor affecting the comparison between molecular and traditional methods is the taxonomic range of bioindicators used. The macroinvertebrate indicator groups used in the calculation of the biotic index are selected due to their known biotic responses to stressors and their size and are not a monophyletic group. In order to target those metazoans traditionally surveyed, the metabarcoding approach relies on highly degenerate primers [65], which amplify a wide range of non-target DNA fragments from eDNA samples. This approach therefore has the drawback of unspecific amplification of non-target organisms at the cost of target organism sequences [27]. Therefore, for the comparison of diversity with

the traditional approach, only a small fraction of the eDNA reads corresponding to macroinvertebrate indicator taxa are used in the biotic index. In combination with the high diversity detected with degenerate primers, this might hamper the detection of less abundant taxa at a site [16], as many reads are assigned to non-target taxa. As a possible solution, local richness measures may be improved by using more recently developed group-specific primers (e.g., [66]) or a combination of multiple markers for multiple groups [67]. With this, the eDNA metabarcoding would be more targeted towards the classical macroinvertebrate indicator taxa without the drawback of amplifying non-target taxa and lost read depth. Alternatively, the taxa considered could be extended and also include the many invertebrates amplified but not considered in the kick-net-based indices.

Inference of the biotic index from eDNA data

Although eDNA unraveled lower indicator taxa richness locally, the overall community composition was similar for both methods and is promising for the implementation of eDNA-based biotic indices for water quality assessments. However, reads from a metabarcoding approach cannot be translated into species counts [29,30], and thus we could not directly calculate the IBCH index based on specimen counts for eDNA reads. As molecular monitoring provides different data than traditional surveys, novel approaches to fully exploit the eDNA derived community assemblages are needed [31,32]. Here, a supervised machine learning algorithm, i.e., random forest [68], was used to predict the biotic index based on the composition of indicator OTUs as predictive features of the ecological state of a site. This data-driven approach allows for the inclusion of the comprehensive list of indicator OTUs, as in contrast to the one-to-one filtering for the diversity measures, it is not based on taxonomically assigned OTUs as features. Instead of restricting the input data to the taxonomic levels of the biotic index (n = 205 OTUs), the machine learning algorithm included

all OTUs belonging to surveyed macroinvertebrate phyla (n = 693 OTUs). That is, the machine learning approach was based on the OTU-level diversity of aquatic communities at a site. By this, we were able to include multiple OTUs previously united at individual family levels, thus describe the community on a more nuanced level and therefore extract information not considered by the traditional index calculation. In the kick-net-based index calculations, only organisms captured by kick-net sampling, identified to family level at best and known to be responsive to stressors are included, whereas the eDNA survey is not restricted to these groups.

The incorporation of the OTU-level information resulted in a high rate of accurate predictions for the river sites, despite the previously described mismatch of local richness pattern based on the taxonomic one-to-one comparison. It showed that the great majority of predictions on the state of the river at a site corresponded with the classic approach, and a maximum of one category divergence between these two methods was observed. Importantly, a mismatch between the two methods does not necessarily mean that the eDNA-based approach is less accurate, as both approaches are proxies (each with their inherent error) of a true state to be estimated.

The random forest model is well-suited to deal with high dimensional data [34] such as the community composition (observed OTUs) at each site. Using the kick-net-based observed ecological state as the response variable, the model is trained on a random subset of sites. After the training phase, it is then applied in orderto infer the ecological state of any site based on the composition community, without the need of pre-assigning indicator values to the OTUs. This opens up the opportunity to use the information of metabarcoding more comprehensively and to shift away from the limited range of previously established indicator taxa. Machine learning as a data-driven approach could thus be used to identify sensitive

taxa that were, due to limitations of the traditional methods, previously out of scope for ecological assessments. However, the predictive power of data-driven approaches is limited by the range of data. In this study, the values of the biotic index for most study sites were centered on the categories of "intermediate" and "good"; fewer observations were available to train models for moderate or very good sites, while none of the sites were classified as "poor". With a better coverage of all the possible categories of the index, the predictive power of the machine-learning algorithm could increase, as the distribution of averaged prediction from regression trees is narrower than the observed range of values [69]. Furthermore, the inference of causal links with random forest is limited. The data-driven predictions and the resulting classifications do not provide a mechanistic understanding of the biotic index calculation. The interpretation of misclassifications relies on the ecological understanding of the importance of input features, i.e. OTUs for the prediction. Despite these limitations, machine learning approaches have shown similar applicability for other biotic indicator taxa such as diatoms [24], macroinvertebrates [70], and taxonomy-free based approaches on prokaryotic and eukaryotic communities [36]. Overall, supervised machine learning can offer complementing or novel insights for the interpretation of big data in an ecological context, especially in the context of biotic indices when the like-for-like comparison is hindered by methodological differences.

Conclusion

This study shows that eDNA sampling from water can be complementary to kick-net sampling in the assessments of macroinvertebrate biodiversity and, more importantly, match indices on the ecological state. By carrying out a comparison of eDNA sampling with kick-net samples on a national routine monitoring scale, we take a crucial step in moving molecular surveys into future monitoring applications. A one-to-one comparison of eDNA

with traditional methods is impossible, due to the inherent differences in the units sampled (DNA vs. specimens). However, molecular surveys offer a different perspective on aquatic communities and eDNA sampling can add to the understanding of the ecological state of rivers, which is an important way forward in the management and conservation of freshwater ecosystems.

Acknowledgments

We thank Silvia Kobel and Aria Minder for technical advice in the laboratory. We thank the Swiss Federal Office for the Environment (BAFU/FOEN) and all the contractors for logistic support and the provision of the eDNA samples. The data analyzed in this paper were generated in collaboration with the Genetic Diversity Centre (GDC), ETH Zurich. Funding for the project (to FA) is from the Swiss National Science Foundation Grant No 31003A_173074 and the Swiss Federal Office for the Environment (BAFU/FOEN).

Author contributions

FA and JB designed the study. Laboratory work was carried out by JB. JC performed the bioinformatics analysis and JB analyzed the data. JB, RB, JC, and FA wrote the manuscript and all authors contributed to revising the text.

Data availability

The raw sequencing data analyzed in this study is open for access in the European Nucleotide Archive with the accession number PRJEB44539.

References

1. Dirzo R, Young HS, Galetti M, Ceballos G, Isaac NJB, Collen B. Defaunation in the Anthropocene. Science. 2014;345: 401–406. doi:10.1126/science.1251817

2. Dudgeon D. Multiple threats imperil freshwater biodiversity in the Anthropocene. Current Biology. 2019;29: 960–967. doi:10.1016/j.cub.2019.08.002

3. Brauman KA, Garibaldi LA, Polasky S, Aumeeruddy-Thomas Y, Brancalion PHS, DeClerck F, et al. Global trends in nature's contributions to people. Proceedings of the National Academy of Sciences. 2020; 202010473. doi:10.1073/pnas.2010473117

4. Karr J. Biological Integrity - a Long-Neglected Aspect of Water-Resource Management. Ecological Applications. 1991;1: 66–84. doi:10.2307/1941848

5. Borja A, Dauer DM, Gremare A. The importance of setting targets and reference conditions in assessing marine ecosystem quality. Ecological Indicators. 2012;12: 1–7. doi:10.1016/j.ecolind.2011.06.018

6. Pawlowski J, Apothéloz-Perret-Gentil L, Altermatt F. Environmental DNA: What's behind the term? Clarifying the terminology and recommendations for its future use in biomonitoring. Molecular Ecology. 2020;29: 4258–4264. doi:https://doi.org/10.1111/mec.15643

7. Barbour M, Gerritsen J, Snyder B, Stribling J. Rapid bioassessment protocols foruse in streams and wadable rivers: Periphyton, benthic invertebrates and fish. Second Edition. U.S. Environmental Protection Agency; Office of Water; Washington, D.C.; 1999. Available: http://www.epa.gov/OWOW/monitoring/techmon.html

8. Lobo EA, Heinrich CG, Schuch M, Wetzel CE, Ector L. Diatoms as Bioindicators in Rivers. In: Necchi JR O, editor. River Algae. Cham: Springer International Publishing; 2016.

pp. 245–271. doi:10.1007/978-3-319-31984-1_11

9. Sweeney BW, Battle JM, Jackson JK, Dapkey T. Can DNA barcodes of stream macroinvertebrates improve descriptions of community structure and water quality? Journal of the North American Benthological Society. 2011;30: 195–216. doi:10.1899/10-016.1

10. Mächler E, Deiner K, Steinmann P, Altermatt F. Utility of environmental DNA for monitoring rare and indicator macroinvertebrate species. Freshwater Science. 2014;33: 1174–1183. doi:10.1086/678128

11. Deiner K, Bik HM, Mächler E, Seymour M, Lacoursière-Roussel A, Altermatt F, et al. Environmental DNA metabarcoding: Transforming how we survey animal and plant communities. Molecular Ecology. 2017;26: 5872–5895. doi:10.1111/mec.14350

12. Altermatt F, Little CJ, Mächler E, Wang S, Zhang X, Blackman RC. Uncovering the complete biodiversity structure in spatial networks: the example of riverine systems. Oikos. 2020;129: 607–618. doi:https://doi.org/10.1111/oik.06806

13. Taberlet P, Coissac E, Pompanon F, Brochmann C, Willerslev E. Towards next-generation biodiversity assessment using DNA metabarcoding. Molecular Ecology. 2012;21: 2045–2050. doi:10.1111/j.1365-294X.2012.05470.x

14. Aglieri G, Baillie C, Mariani S, Cattano C, Calò A, Turco G, et al. Environmental DNA effectively captures functional diversity of coastal fish communities. Molecular Ecology. 00: 1–13. doi:https://doi.org/10.1111/mec.15661

15. Pont D, Valentini A, Rocle M, Maire A, Delaigue O, Jean P, et al. The future of fish-based ecological assessment of European rivers: from traditional EU Water Framework Directive compliant methods to eDNA metabarcoding-based approaches. Journal of Fish Biology. 2019;98: 354–366. doi:https://doi.org/10.1111/jfb.14176

16. Carew ME, Pettigrove VJ, Metzeling L, Hoffmann AA. Environmental monitoring using

next generation sequencing: rapid identification of macroinvertebrate bioindicator species. Frontiers in Zoology. 2013;10: 45. doi:10.1186/1742-9994-10-45

17. Fernández S, Rodríguez S, Martínez JL, Borrell YJ, Ardura A, García-Vázquez E. Evaluating freshwater macroinvertebrates from eDNA metabarcoding: A river Nalón case study. PLOS ONE. 2018;13: e0201741. doi:10.1371/journal.pone.0201741

18. Uchida N, Kubota K, Aita S, Kazama S. Aquatic insect community structure revealed by eDNA metabarcoding derives indices for environmental assessment. PeerJ. 2020;8: e9176. doi:10.7717/peerj.9176

19. Camargo JA. Responses of aquatic macrophytes to anthropogenic pressures: comparison between macrophyte metrics and indices. Environmental Monitoring and Assessment. 2018;190: 173. doi:10.1007/s10661-018-6549-y

20. Ortega A, Geraldi NR, Duarte CM. Environmental DNA identifies marine macrophyte contributions to Blue Carbon sediments. Limnology and Oceanography. 2020;65: 3139–3149. doi:https://doi.org/10.1002/lno.11579

21. Apothéloz-Perret-Gentil L, Bouchez A, Cordier T, Cordonier A, Guéguen J, Rimet F, et al. Monitoring the ecological status of rivers with diatom eDNA metabarcoding: A comparison of taxonomic markers and analytical approaches for the inference of a molecular diatom index. Molecular Ecology. 2020;00: 1–10. doi:https://doi.org/10.1111/mec.15646

22. Rimet F, Abarca N, Bouchez A, Kusber W-H, Jahn R, Kahlert M, et al. The potential of High-Throughput Sequencing (HTS) of natural samples as a source of primary taxonomic information for reference libraries of diatom barcodes. Fottea. 2018;18: 37–54. doi:10.5507/fot.2017.013

23. Li F, Peng Y, Fang W, Altermatt F, Xie Y, Yang J, et al. Application of Environmental

DNA Metabarcoding for Predicting Anthropogenic Pollution in Rivers. Environmental Science & Technology. 2018;52: 11708–11719. doi:10.1021/acs.est.8b03869

24. Pawlowski J, Kelly-Quinn M, Altermatt F, Apothéloz-Perret-Gentil L, Beja P, Boggero A, et al. The future of biotic indices in the ecogenomic era: Integrating (e)DNA metabarcoding in biological assessment of aquatic ecosystems. Science of The Total Environment. 2018;637–638: 1295–1310. doi:10.1016/j.scitotenv.2018.05.002

25. Bush A, Compson ZG, Monk WA, Porter TM, Steeves R, Emilson E, et al. Studying Ecosystems With DNA Metabarcoding: Lessons From Biomonitoring of Aquatic Macroinvertebrates. Frontiers in Ecology and Evolution. 2019;7: 434. doi:10.3389/fevo.2019.00434

26. Carew ME, Miller AD, Hoffmann AA. Phylogenetic signals and ecotoxicological responses: potential implications for aquatic biomonitoring. Ecotoxicology. 2011;20: 595–606. doi:10.1007/s10646-011-0615-3

27. Hajibabaei M, Porter T, Robinson C, Baird D, Shokralla S, Wright M. Watered-down biodiversity? A comparison of metabarcoding results from DNA extracted from matched water and bulk tissue biomonitoring samples. 2019. doi:10.1101/575928

28. Deiner K, Fronhofer EA, Mächler E, Walser J-C, Altermatt F. Environmental DNA reveals that rivers are conveyer belts of biodiversity information. Nature Communications. 2016;7: 12544. doi:10.1038/ncomms12544

29. Elbrecht V, Leese F. Can DNA-Based Ecosystem Assessments Quantify Species Abundance? Testing Primer Bias and Biomass—Sequence Relationships with an Innovative Metabarcoding Protocol. Hajibabaei M, editor. PLoS ONE. 2015;10: e0130324. doi:10.1371/journal.pone.0130324

30. Piñol J, Senar MA, Symondson WOC. The choice of universal primers and the

characteristics of the species mixture determine when DNA metabarcoding can be quantitative. Molecular Ecology. 2019;28: 407–419. doi:https://doi.org/10.1111/mec.14776

31. Garcia C. From ecological indicators to ecological functioning: Integrative approaches to seize on ecological, climatic and socio-economic databases. Ecological Indicators. 2019;107: 105612. doi:10.1016/j.ecolind.2019.105612

32. Compson ZG, McClenaghan B, Singer GAC, Fahner NA, Hajibabaei M. Metabarcoding From Microbes to Mammals: Comprehensive Bioassessment on a Global Scale. Frontiers in Ecology and Evolution. 2020;8. doi:10.3389/fevo.2020.581835

33. Jordan MI, Mitchell TM. Machine learning: Trends, perspectives, and prospects. Science. 2015;349: 255–260. doi:10.1126/science.aaa8415

34. Cutler A, Cutler D, Stevens J. Random Forests. Machine Learning - ML. 2011. pp. 157–176. doi:10.1007/978-1-4419-9326-7_5

35. Bohan DA, Vacher C, Tamaddoni-Nezhad A, Raybould A, Dumbrell AJ, Woodward G. Next-Generation Global Biomonitoring: Large-scale, Automated Reconstruction of Ecological Networks. Trends in Ecology & Evolution. 2017;32: 477–487. doi:10.1016/j.tree.2017.03.001

36. Cordier T, Forster D, Dufresne Y, Martins C, Stoeck T, Pawlowski J. Supervised machine learning outperforms taxonomy-based environmental DNA metabarcoding applied to biomonitoring. Molecular Ecology Resources. 2018;18. doi:10.1111/1755-0998.12926

37. Keck F, Vasselon V, Rimet F, Bouchez A, Kahlert M. Boosting DNA metabarcoding for biomonitoring with phylogenetic estimation of operational taxonomic units' ecological profiles. Molecular Ecology Resources. 2018;18: 1299–1309. doi:https://doi.org/10.1111/1755-0998.12919

38. Frühe L, Cordier T, Dully V, Breiner H, Lentendu G, Pawlowski J, et al. Supervised machine learning is superior to indicator value inference in monitoring the environmental

impacts of salmon aquaculture using eDNA metabarcodes. Molecular Ecology. 2020;00: 1–19. doi:10.1111/mec.15434

39. FOEN, Bundesamt für Umwelt BAFU | Office fédéral de l’environnement OFEV | Ufficio federale dell’ambiente. NAWA – Nationale Beobachtung Oberflächengewässerqualität. Konzept Fliessgewässer. Umwelt-Wissen. 2013;Nr. 1327: 72 S.

40. Pawlowski J, Apothéloz-Perret-Gentil L, Mächler E, Altermatt F. Environmental DNA applications in biomonitoring and bioassessment of aquatic ecosystems. Guidelines. Federal Office for the Environment, Bern. Environmental Studies. 2020; 71 S.

41. Stucki P. Methoden zur Untersuchung und Beurteilung der Fliessgewässer: Makrozoobenthos Stufe F. Bundesamt für Umwelt, Bern Umwelt-Vollzug. 2010; Umwelt-Vollzug Nr. 1026: 61 S.

42. Deiner K, Walser J-C, Mächler E, Altermatt F. Choice of capture and extraction methods affect detection of freshwater biodiversity from environmental DNA. Biological Conservation. 2015;183: 53–63. doi:10.1016/j.biocon.2014.11.018

43. Leray M, Yang JY, Meyer CP, Mills SC, Agudelo N, Ranwez V, et al. A new versatile primer set targeting a short fragment of the mitochondrial COI region for metabarcoding metazoan diversity: application for characterizing coral reef fish gut contents. Frontiers in Zoology. 2013;10: 34. doi:10.1186/1742-9994-10-34

44. Geller J, Meyer C, Parker M, Hawk H. Redesign of PCR primers for mitochondrial cytochrome c oxidase subunit I for marine invertebrates and application in all-taxa biotic surveys. Molecular Ecology Resources. 2013;13: 851–861. doi:10.1111/1755-0998.12138

45. Andrews S, Krueger F, Segonds-Pichon A, Biggins L, Krueger C, Wingett S. FastQC A Quality Control tool for High Throughput Sequence Data. Babraham Bioinformatics. 2012. Available: https://www.bioinformatics.babraham.ac.uk/projects/fastqc/

46. R Core Team. R: A language and environment for statistical computing. Available: https://www.R-project.org/

47. McMurdie PJ, Holmes S. phyloseq: An R Package for Reproducible Interactive Analysis and Graphics of Microbiome Census Data. PLoS ONE. 2013;8: e61217. doi:10.1371/journal.pone.0061217

48. Alther R, Altermatt F. SwissRiverPlot: Package to plot the Swiss river network in a customizable way. https://github.com/romanalther. 2020;R package version 0.2-14.

49. Brunson CJ. ggalluavial: Alluvial Plots in ggplot2. In: R package ggalluvial. 2020. Available: https://cran.r-project.org/web/packages/ggalluvial/vignettes/ggalluvial.html

50. Wickham H, Chang W, Henry L, Pedersen TL, Takahashi K, Wilke C, et al. ggplot2: Create Elegant Data Visualisations Using the Grammar of Graphics. 2020. Available: https://CRAN.R-project.org/package=ggplot2

51. Kuhn M, Wing J, Weston S, Williams A, Keefer C, Engelhardt A, et al. caret: Classification and Regression Training. 2020. Available: https://CRAN.R-project.org/package=caret

52. Wright MN, Ziegler A. ranger: A Fast Implementation of Random Forests for High Dimensional Data in C++ and R. Journal of Statistical Software. 2017;77. doi:10.18637/jss.v077.i01

53. Cohen J. A Coefficient of Agreement for Nominal Scales. Educational and Psychological Measurement. 1960;20: 37–46. doi:10.1177/001316446002000104

54. Macher J-N, Vivancos A, Piggott JJ, Centeno FC, Matthaei CD, Leese F. Comparison of environmental DNA and bulk-sample metabarcoding using highly degenerate cytochrome c oxidase I primers. Molecular Ecology Resources. 2018;18: 1456–1468. doi:10.1111/1755-0998.12940

55. Mächler E, Little CJ, Wüthrich R, Alther R, Fronhofer EA, Gounand I, et al. Assessing different components of diversity across a river network using eDNA. Environmental DNA. 2019;1: 290–301. doi:https://doi.org/10.1002/edn3.33

56. Leese F, Sander M, Buchner D, Elbrecht V, Haase P, Zizka VMA. Improved freshwater macroinvertebrate detection from environmental DNA through minimized nontarget amplification. Environmental DNA. 2021;3: 261–276. doi:10.1002/edn3.177

57. Gleason JE, Elbrecht V, Braukmann TWA, Hanner RH, Cottenie K. Assessment of stream macroinvertebrate communities with eDNA is not congruent with tissue-based metabarcoding. Molecular Ecology. 2020;00: 1–13. doi:10.1111/mec.15597

58. Allan EA, Zhang WG, Lavery AC, Govindarajan AF. Environmental DNA shedding and decay rates from diverse animal forms and thermal regimes. Environmental DNA. 2021;3: 492–514. doi:https://doi.org/10.1002/edn3.141

59. Deiner K, Altermatt F. Transport Distance of Invertebrate Environmental DNA in a Natural River. PLOS ONE. 2014;9: e88786. doi:10.1371/journal.pone.0088786

60. Pont D, Rocle M, Valentini A, Civade R, Jean P, Maire A, et al. Environmental DNA reveals quantitative patterns of fish biodiversity in large rivers despite its downstream transportation. Sci Rep. 2018;8: 10361. doi:10.1038/s41598-018-28424-8

61. Carraro L, Mächler E, Wüthrich R, Altermatt F. Environmental DNA allows upscaling spatial patterns of biodiversity in freshwater ecosystems. Nature Communications. 2020;11: 3585. doi:10.1038/s41467-020-17337-8

62. Dickie IA, Boyer S, Buckley HL, Duncan RP, Gardner PP, Hogg ID, et al. Towards robust and repeatable sampling methods in eDNA-based studies. Molecular Ecology Resources. 2018;18: 940–952. doi:https://doi.org/10.1111/1755-0998.12907

63. Carraro L, Stauffer JB, Altermatt F. How to design optimal eDNA sampling strategies

for biomonitoring in river networks. Environmental DNA. 2021;3: 157–172. doi:10.1002/edn3.137

64. Altermatt F. Diversity in riverine metacommunities: a network perspective. Aquatic Ecology. 2013;47: 365–377. doi:10.1007/s10452-013-9450-3

65. Elbrecht V, Leese F. Validation and Development of COI Metabarcoding Primers for Freshwater Macroinvertebrate Bioassessment. Frontiers in Environmental Science. 2017;5: 11. doi:10.3389/fenvs.2017.00011

66. Meyer A, Boyer F, Valentini A, Bonin A, Ficetola GF, Beisel J-N, et al. Morphological vs. DNA metabarcoding approaches for the evaluation of stream ecological status with benthic invertebrates: Testing different combinations of markers and strategies of data filtering. Molecular Ecology. 2020;00: 1–18. doi:https://doi.org/10.1111/mec.15723

67. Hajibabaei M, Porter TM, Wright M, Rudar J. COI metabarcoding primer choice affects richness and recovery of indicator taxa in freshwater systems. PLOS ONE. 2019;14: e0220953. doi:10.1371/journal.pone.0220953

68. Breiman L. Random Forests. Machine Learning. 2001;45: 5–32. doi:10.1023/A:1010933404324

69. Zhang X. Environmental DNA Shaping A New Era of Ecotoxicological Research. Environmental Science & Technology. 2019 [cited 29 Apr 2019]. doi:10.1021/acs.est.8b06631

70. Fan J, Wang S, Li H, Yan Z, Zhang Y, Zheng X, et al. Modeling the ecological status response of rivers to multiple stressors using machine learning: A comparison of environmental DNA metabarcoding and morphological data. Water Research. 2020;183: 116004. doi:10.1016/j.watres.2020.116004

Figure 1 Click here to access/download;Figure;Fig 1.eps

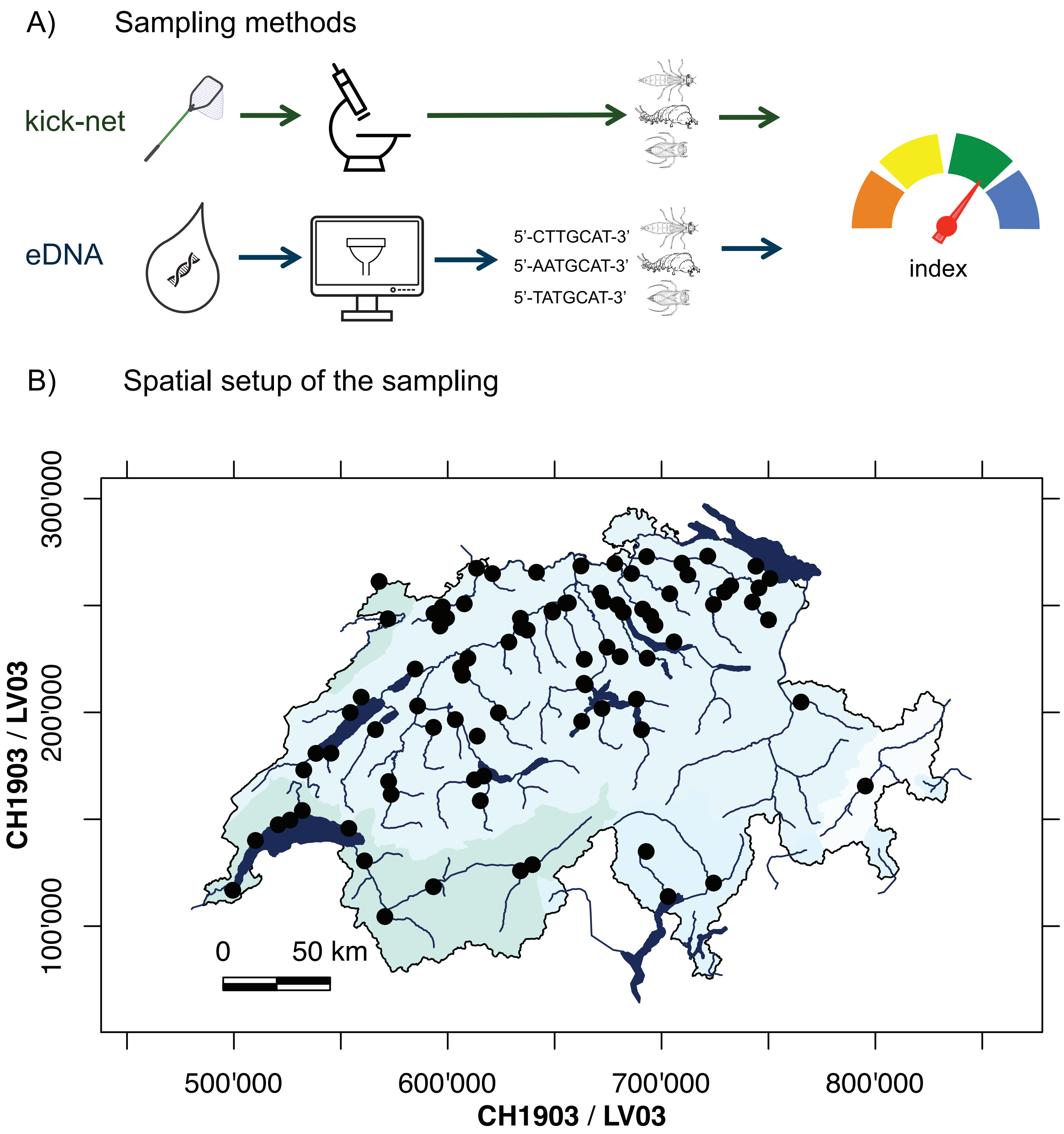

Figure 2

Click here to access/download;Figure;Fig 2.eps

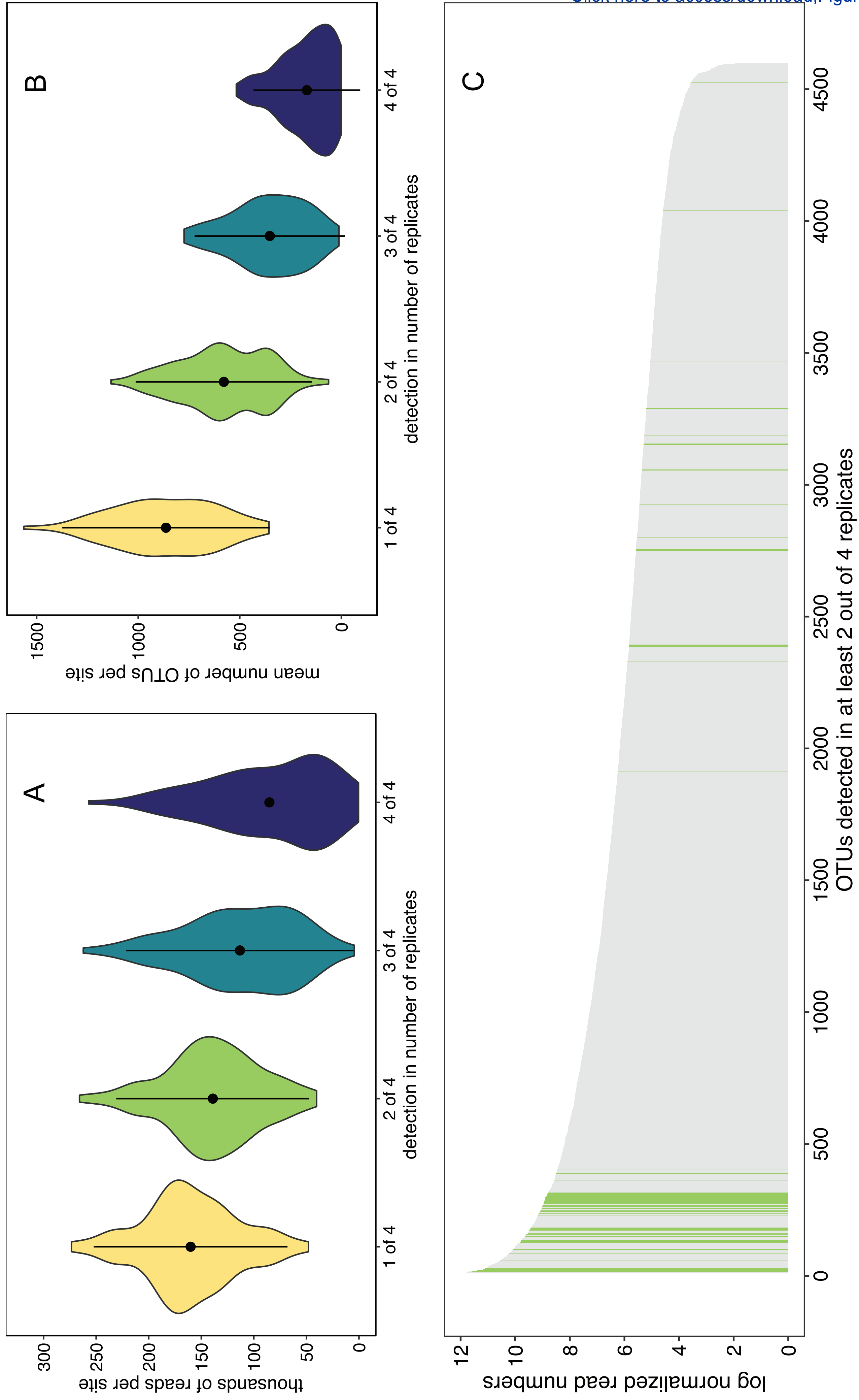

Figure 3

Click here to access/download;Figure;Fig 3.eps

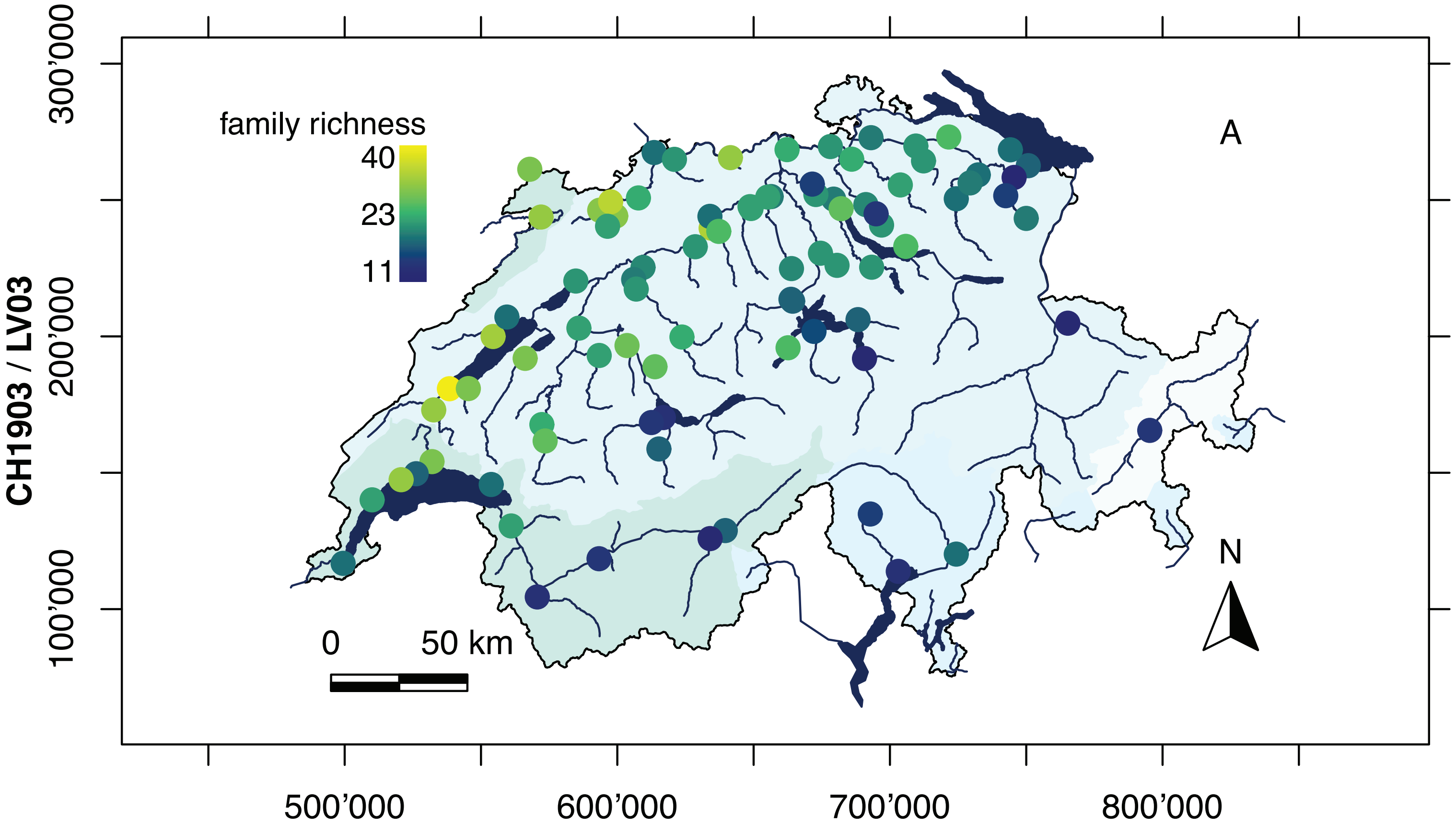

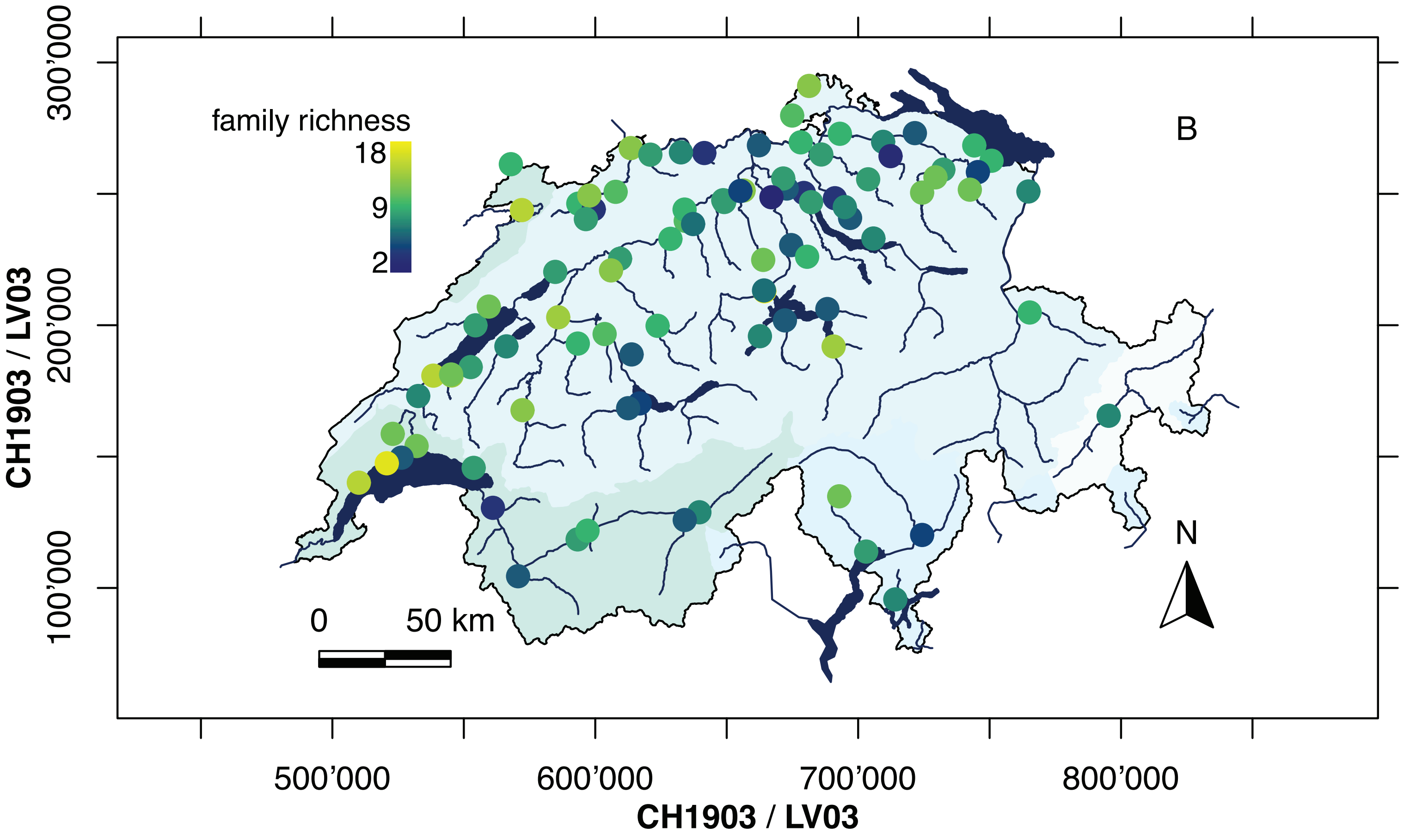

Figure 4 Click here to access/download;Figure;Fig 4.eps

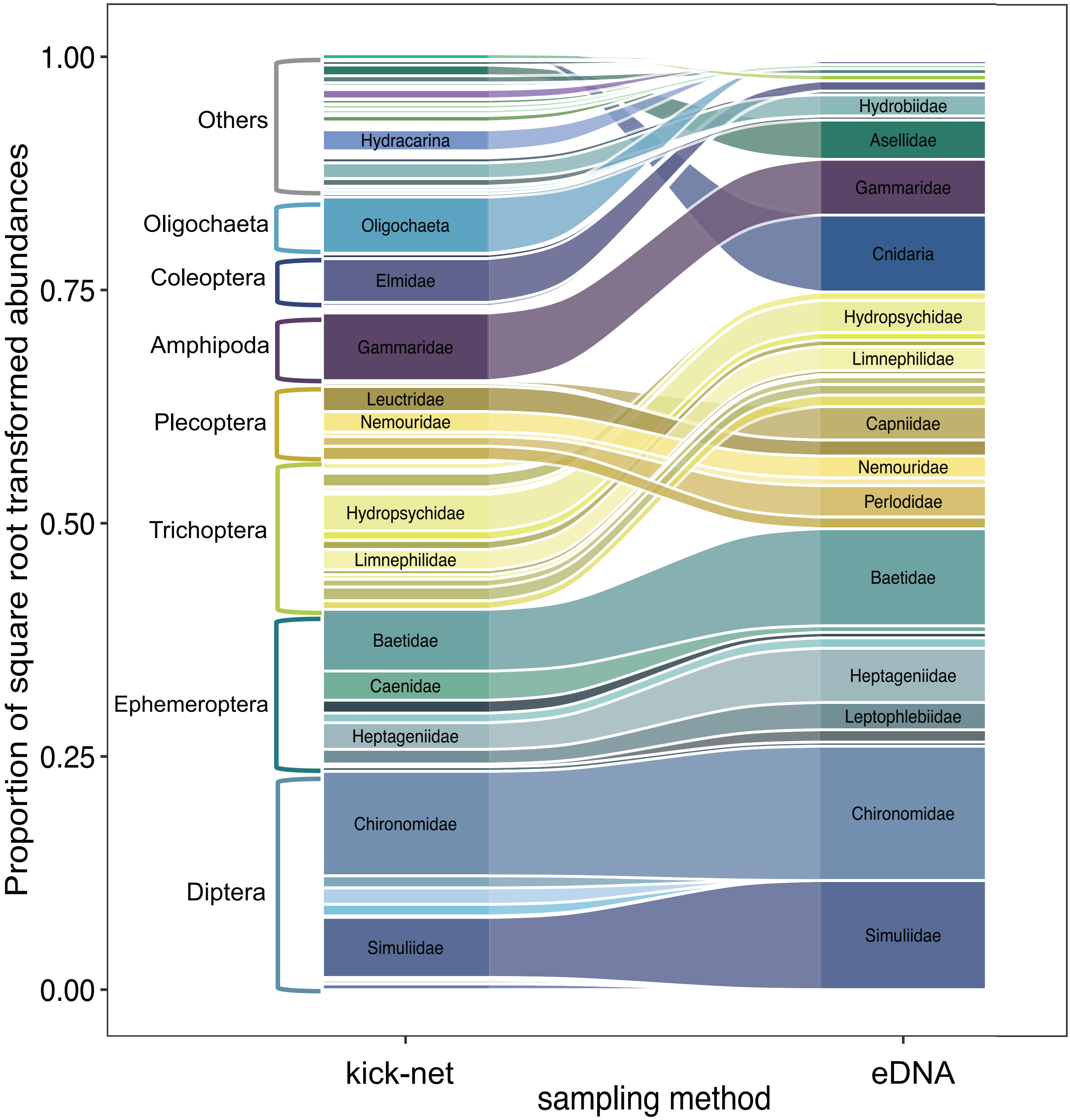

Figure 5 Click here to access/download;Figure;Fig 5.eps

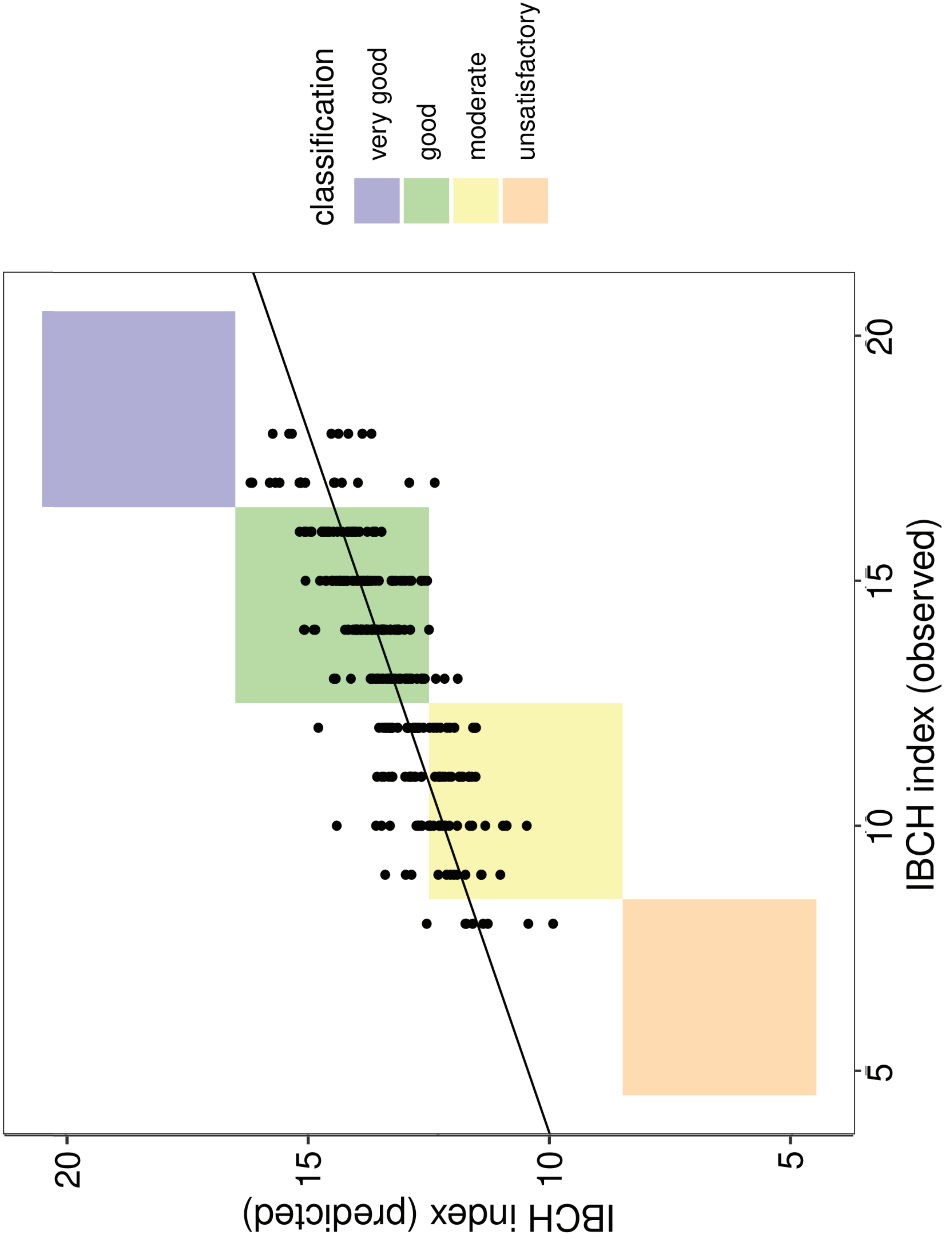

Figure 6

Click here to access/download;Figure;Fig 6.eps

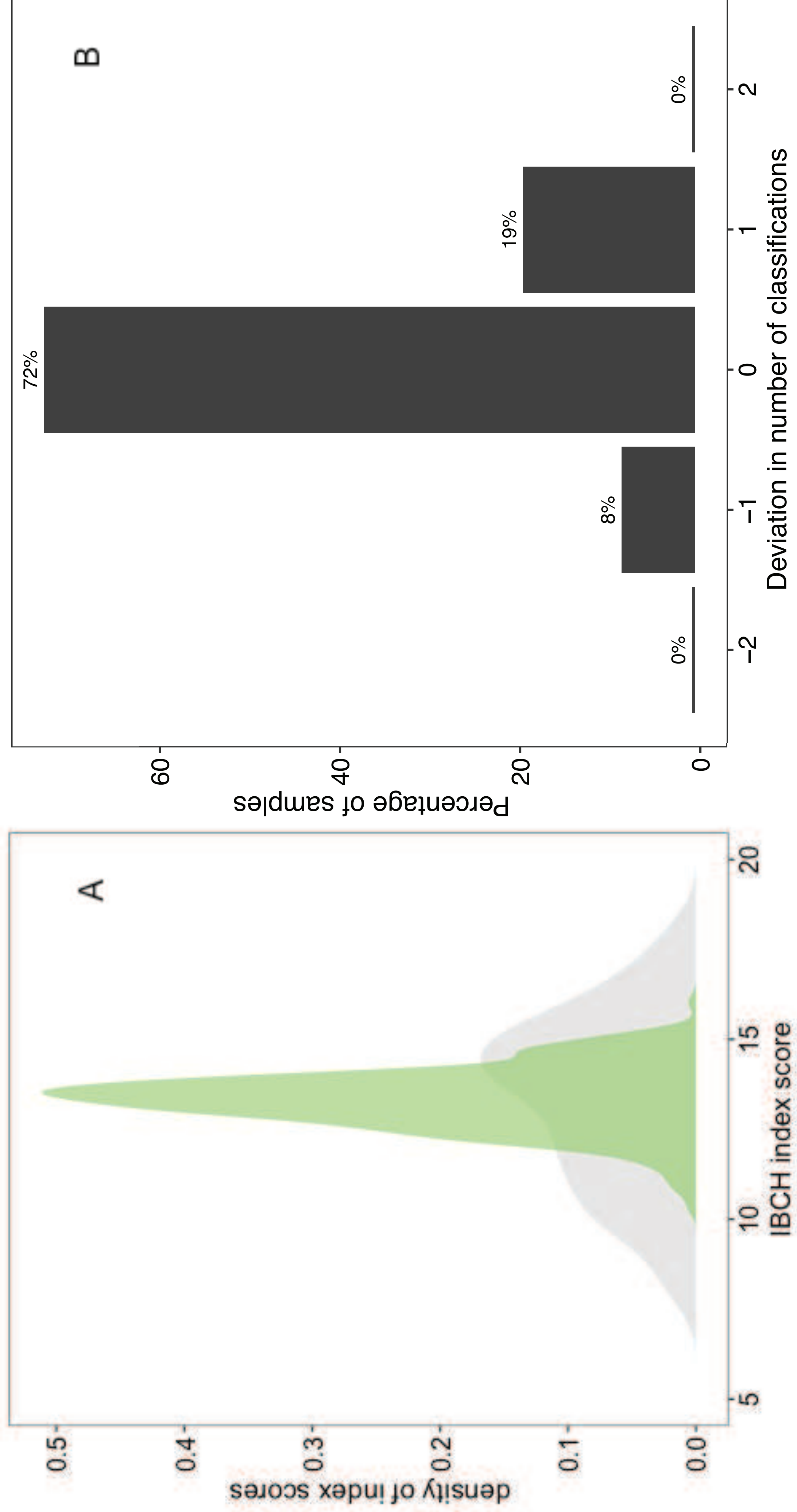

Click here to access/download
Supporting Information
SupportingInformation_Brantschen_etal.docx
